# The sensitivity of diffusion MRI to microstructural properties and experimental factors

**DOI:** 10.1016/j.jneumeth.2020.108951

**Published:** 2021-01-01

**Authors:** Maryam Afzali, Tomasz Pieciak, Sharlene Newman, Eleftherios Garyfallidis, Evren Özarslan, Hu Cheng, Derek K Jones

**Affiliations:** a Cardiff University Brain Research Imaging Centre (CUBRIC), School of Psychology, Cardiff University, Cardiff, United Kingdom; b AGH University of Science and Technology, Kraków, Poland; c LPI, ETSI Telecomunicación, Universidad de Valladolid, Valladolid, Spain; d Department of Psychological and Brain Sciences, Indiana University, Bloomington, IN 47405, USA; e Program of Neuroscience, Indiana University, Bloomington, IN 47405, USA; f Department of Intelligent Systems Engineering, Indiana University, Bloomington, IN 47408, USA; g Department of Biomedical Engineering, Linköping University, Linköping, Sweden; h Center for Medical Image Science and Visualization, Linköping University, Linköping, Sweden

**Keywords:** Diffusion MRI, Signal representation, Biophysical model, Microstructure, Experimental factors, Anisotropy

## Abstract

Diffusion MRI is a non-invasive technique to study brain microstructure. Differences in the microstructural properties of tissue, including size and anisotropy, can be represented in the signal if the appropriate method of acquisition is used. However, to depict the underlying properties, special care must be taken when designing the acquisition protocol as any changes in the procedure might impact on quantitative measurements. This work reviews state-of-the-art methods for studying brain microstructure using diffusion MRI and their sensitivity to microstructural differences and various experimental factors. Microstructural properties of the tissue at a micrometer scale can be linked to the diffusion signal at a millimeter-scale using modeling. In this paper, we first give an introduction to diffusion MRI and different encoding schemes. Then, signal representation-based methods and multi-compartment models are explained briefly. The sensitivity of the diffusion MRI signal to the microstructural components and the effects of curvedness of axonal trajectories on the diffusion signal are reviewed. Factors that impact on the quality (accuracy and precision) of derived metrics are then reviewed, including the impact of random noise, and variations in the acquisition parameters (i.e., number of sampled signals, *b*-value and number of acquisition shells). Finally, yet importantly, typical approaches to deal with experimental factors are depicted, including unbiased measures and harmonization. We conclude the review with some future directions and recommendations on this topic.

## Highlights

• This work reviews different methods for studying brain microstructure using dMRI.
• Sensitivity to microstructural differences and experimental factors is investigated.
• Signal representation-based methods and multi-compartment models are explained.

## 1 Introduction

The classical way of studying microstructural information of tissue is histology. This method has some limitations; it needs a biopsy, tissue preparation, the samples are small, and longitudinal measurements of the same sample are not easy (Gurcan et al., 2009). Diffusion MRI, on the other hand, can provide information about tissue microstructure non-invasively (Le Bihan et al., 1988). The advantages of the technique compared to histology are that it does not need a biopsy or tissue preparation, is a non-invasive technique, and is easy to run repeated measurements (Basser et al., 1994b, Le Bihan, 2003, Jones, 2010). The imaging field-of-view can be large enough to cover the whole organ instead of imaging only a small sample of the tissue. The data acquisition is faster than analysis of histology sections (Alexander et al., 2019).

Histological studies have provided a lot of knowledge about brain microstructure and connectivity (Braak and Braak, 1995). The very first works in this area began with post-mortem tissue (Schulz et al., 1980). The non-invasive nature of diffusion MRI makes it feasible to study brain microstructure in healthy volunteers as well as patients (van Gelderen et al., 1994, Fazekas et al., 1987, Shenton et al., 1992). The acquisition of data on a population is possible and therefore group analysis studies are feasible (Afzali et al., 2011). It is also possible to make repeated measurements in the study of brain development or *ex-vivo* studies (Roebroeck et al., 2019) and in pathological disorders, preventing the risk and side-effects of the biopsy (Kreth et al., 2001). The study of the whole organ is possible preventing the false-negative effect due to sampling the wrong part of the tissue.

The level of anatomical detail in histology studies is much higher than in microstructure imaging techniques. The submicron resolution in histology/electron microscopy provides insight into the cellular structure of the tissue while the millimeter resolution of diffusion MRI provides statistical descriptions of the tissue. In some cancer studies, information at the cellular level is useful while in some other biomedical applications, being able to detect statistical changes in the tissue is useful (Mouras et al., 2010). For example, the size distribution of axons in white matter determines the conduction velocity (Drakesmith et al., 2019). Different shapes and configurations of the cells can indicate the type of tumor (Kauppinen, 2002).

Diffusion MRI provides a tool to study brain tissue based on the Brownian motion of water molecules (Tanner, 1979; Le Bihan et al., 1988) and it is therefore sensitive to differences in the microstructure of the tissue (Callaghan et al., 1988, Basser et al., 1994b, Jones, 2010). In this technique, the images are acquired with different number of directions, *b*-values, *b*-tensor encoding schemes (Callaghan et al., 1988, Basser et al., 1994b, Jones, 2010, Westin et al., 2016). Then a model is fitted to the signal and a set of parameters can be obtained for each voxel in the image—either for signal representations (e.g. DT-MRI) (Basser et al., 1994b) or modelling (Stanisz et al., 1997). These parameters are related to the microstructural properties of the tissue. Diffusion MRI sensitizes the signal to the random motion of the water molecules in a diffusion time from millisecond up to one second. At room or body temperature, the mean displacement due to motion over this time-scale is at the scale of the micrometer, which is the cellular scale. Therefore the cellular structure of the tissue directly affects the motion of the water molecules, so diffusion MRI is a useful tool to study the tissue microstructure.

Diffusion MRI has found a lot of applications in biomedical imaging in recent years. This work reviews the sensitivity of the diffusion signal to the microstructure of the underlying tissue and the experimental factors. Therefore, we focus on signal representation techniques as well as biophysical modeling (Novikov et al., 2019). In this review, we will explain how the signal is sensitive to the underlying microstructure and how it can be misinterpreted in the presence of noise and various experimental factors. In Section 2 we explain brain microstructure briefly. In Section 3 we provide some background information about the diffusion MRI signal and different encoding schemes. In Section 4 we focus on diffusion signal representation-based methods and then in Section 5 we introduce multi-compartment models, the sensitivity of the signal to the axon diameter, size distribution, and curvedness of neural trajectories. Furthermore, we present the limitations of multi-compartment models and present the inherent effects of model fitting procedures. Next, in Section 6, we explain the effect of noise and present typical experimental factors that might affect diffusion MRI studies. Finally, in the last section, we conclude the review and give some hints and tips for future directions.

## 2 Brain microstructure

The brain contains neurons and glial cells and has three main parts; white matter (WM), gray matter (GM), and cerebrospinal fluid (CSF). The gray matter includes cell bodies and dendrites. White matter is mainly composed of densely packed axons that emerge from the soma in the GM. Glial cells are also present in the WM (Buzsáki et al., 2013). The diameter of dendrites is around 0.2–3 μm. The structure of the dendritic branches depends on the type of neuron (Fiala et al., 2007). A few dendrites (less than five) emerge directly from the soma. In the cerebral cortex the branches of the dendritic tree are isotropically distributed while in other regions such as the hippocampus, they are anisotropic (Zaqout and Kaindl, 2016). The length of axons in the human brain changes from a few millimeters in intra-cortical connections to around 1 meter in the corticospinal pathway (Schüz and Braitenberg, 2002). In the WM the axon diameter ranges from 0.1 to 10 μm (Aboitiz et al., 1992, LaMantia and Rakic, 1990, Ong et al., 2008, Waxman and Bennett, 1972, Drakesmith et al., 2019). The axon diameter distribution (ADD) is different in different species (Innocenti et al., 2014, Innocenti et al., 2016, Caminiti et al., 2009). When modeling axon diameters, a gamma distribution is normally assumed for this distribution (Sepehrband et al., 2016a, Assaf et al., 2008) though alternative distributions have been considered based on optimal information flow subject to relevant constraints (Pajevic and Basser, 2013). The mean of the ADD containing the myelinated axons is around 0.5–0.8 μm. Most of the diffusion-based ADD measurements, to date, have been made in the mid-sagittal corpus callosum (CC). The reason is that the fibers in the callosum are most co-aligned and this makes the orientation known and reduces the complexity of the modeling. The mid-body has a larger mean ADD than the genu, the smallest ADD in CC is observed in splenium (Aboitiz et al., 1992, LaMantia and Rakic, 1990, Caminiti et al., 2009, Riise and Pakkenberg, 2011). In mammals, the brain connection through the mid-body has larger axons (Caminiti et al., 2009).

The myelin contains 80% lipids and 20% proteins with 10 nm thickness wrapping the axons. The myelin divides into segments, the spaces between the segments are the nodes of Ranvier. The length of segments is around 0.2–2 mm (Rushton, 1951) while the length of nodes is 1–2 μm (Salzer, 1997). The myelin increases the conduction velocity (Waxman and Bennett, 1972, Rushton, 1951). The inner diameter of the myelinated axon to the outer diameter of the myelinated axon is called g-ratio and in normal CNS it is around 0.7 (Smith and Koles, 1970). In the CC most of the axons are myelinated. In the genu, the amount of unmyelinated axons is around 16–20% (Aboitiz et al., 1992, LaMantia and Rakic, 1990). The central nervous system contains glial cells. In human adult, the glial cells fall into three categories: 76.6 % oligodendrocytes, 17.3% astrocytes, and 6.5% microglia in number (Pelvig et al., 2008, Salvesen et al., 2017). Oligodendrocytes create the myelin sheaths around axons (Baumann and Pham-Dinh, 2001). Astrocytes have somas with a diameter around 10 μm which generates a star-shaped structure. They are responsible for tissue repair and balancing the amount of extracellular ions (Ding et al., 2016). Microglia provide the first reaction after an injury (see Gehrmann et al., 1995) and their soma is approximately 10 μm in diameter.

In the white matter, there are intra-axonal and extra-cellular spaces. The intra-axonal space is the space-separated by the membrane of axons. In axons, filaments preserve the shape and organization of axons and provide support for the intra-axonal transportation system. Microtubules are part of the cytoskeleton and they aid in transportation. The diameter of the microtubules is around 25 nm. The density of microtubules is related to the axon diameter (Fadić et al., 1985).

In addition to intra-axonal space, there is also extra-cellular space that surrounds cells, axons, and dendrites. The amount of extracellular volume fraction in the non-human brain is reported as 15–35% using invasive microscopy (Syková and Nicholson, 2008). But the shrinkage effect of this microscopy has been reported as 1–65% (Aboitiz et al., 1992, LaMantia and Rakic, 1990, Riise and Pakkenberg, 2011, Houzel et al., 1994).

The images acquired using diffusion MRI are at the scale of mm while the features that we are interested in such as anisotropy, cell size, and axon diameter are at the scale of the micrometer. Each voxel may contain hundreds of thousands of axons that may bend, fan, or cross which makes the modeling of the geometry of the axons complicated.

## 3 Different acquisition schemes

**Fig. 1:**
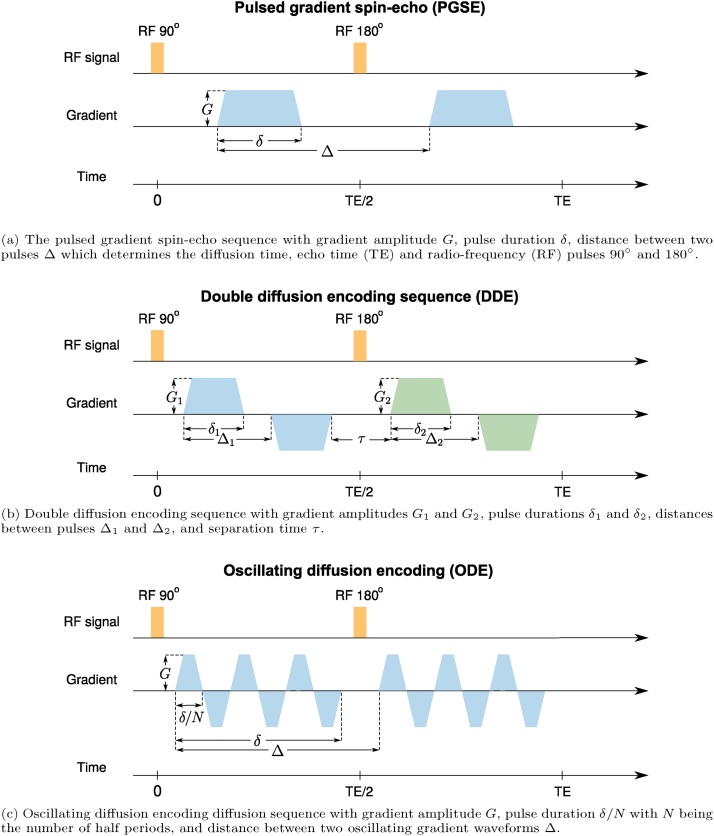
Diffusion acqusition schemes: (a) pulsed gradient spin-echo (PGSE), double diffusion encoding sequence (DDE) and oscillating diffusion encoding (ODE). For more details about diffusion MRI sequences see Table 1 and Section 3.

Time-varying magnetic field gradients are incorporated into MR pulse sequences for encoding diffusion. The most commonly used scheme was introduced by Stejskal and Tanner (1965), which employs a pair of pulsed magnetic field gradients around the 180^∘^ radiofrequency pulse in a spin-echo measurement as illustrated in Fig. 1a. We adopt the nomenclature in Shemesh et al. (2016) and refer to such a Pulsed Gradient Spin Echo (PGSE) sequence by single diffusion encoding (SDE). It should be noted, however, that the method can be incorporated into sequences other than spin-echo based ones. Since its introduction (Stejskal and Tanner, 1965), there have been different works aimed at maximizing the information that can be obtained from a dMRI experiment by exploring different acquisition protocols (Jones, 2004, Alexander, 2008, Alexander et al., 2019).

In SDE, the MR signal is sensitized to diffusion using a pair of gradient pulses that encode the position of the spins along the axis defined by the diffusion gradients. In this sequence, the magnetic field gradient is applied in the direction **g** where the pulse duration is *δ* and the time between the leading edges of the two pulses is Δ which determines the diffusion time (see Fig. 1a). The diffusion of water molecules between and during the pulses attenuates the signal. This attenuation increases when the molecules have a larger displacement between the two pulses.

For free diffusion, the diffusion coefficient can be estimated directly from the signal attenuation based on *δ*, Δ, and gradient strength. In restricted diffusion, however, the displacement is limited and the signal attenuation is smaller than that for free diffusion. The signal attenuation in a restricted medium depends on the size and shape of the pore as well as the parameters of the sequence such as *δ*, Δ, and *G* (gradient strength). By varying the experimental parameters one attempts to obtain the geometric features of the pore (Assaf et al., 2000, Stanisz et al., 1997, Åslund et al., 2009). These parameters are typically collapsed into the so-called the *b*-value. The *b*-value determines the diffusion weighting of a sequence and for SDE is given by *b* = *γ^2^δ^2^G*^2^(Δ − *δ*/3) (Stejskal and Tanner, 1965), where *γ* is the gyromagnetic ratio. The ramp time of the pulse is neglected here. For free diffusion, the *b*-value is sufficient to determine the signal attenuation, which is not sensitive to changes in the timing parameters of the signal as long as they generate the same *b*-value. For free diffusion, the signal exhibits a monoexponential decay according to

(1) S(b)=S(0)exp(−bD),

where *D* is the (scalar) diffusion coefficient. In the presence of restricted diffusion, the signal attenuation depends on the timing parameters of the sequence even though they might provide the same *b*-value.

Some other types of SDE can provide some practical benefits. For example, using asymmetric gradients or twice-refocused spin-echo sequences can reduce the effect of eddy currents (Reese et al., 2003, Finsterbusch, 2009a). Using pulsed-gradient stimulated-echo sequences (PGStE) (Callaghan, 1991) provides longer diffusion times compared to the pulsed gradient spin-echo (PGSE) sequence at the cost of half the signal-to-noise ratio (SNR). In PGSE, the minimum echo time (TE), and therefore minimum *T*_2_-signal loss, is dictated by the diffusion time and the duration of the gradients. PGSE contains a 90° and a 180° radio-frequency pulse while PGStE has three 90° pulses to excite, store and recall the magnetization (Tanner, 1970, Merboldt et al., 1985). In PGStE, only *T*_1_ relaxation occurs between the second and the third 90° pulses, which is typically slower than the *T*_2_ relaxation that attenuates the PGSE signal. Therefore, PGStE can provide a larger signal amplitude at a longer diffusion time. The limitation of PGStE is that half of the SNR is lost in the storage and recall process (Callaghan, 1991, Schick, 1998). Therefore, PGStE is preferred over PGSE when long diffusion times are desired (Özarslan et al., 2012) and for tissue in which the *T*_2_ is relatively short (e.g., muscle). Another type of SDE employs gradient pulses of different durations, providing sensitivity to the pore shape (Laun et al., 2011).

In addition to the size and shape of the cells, other properties such as exchange, intra-voxel incoherent motion (IVIM), and fiber density are among the quantities and phenomena that influence the signal. Each voxel may contain several compartments, including cell bodies, intra-axonal and extra-cellular spaces, and glial cells. Using multi-shell acquisitions, one may be able to extract the anisotropy and density of different compartments (Kaden et al., 2007, Jespersen et al., 2007). The size of the compartments can be estimated by changing the diffusion time in SDE acquisitions (Callaghan, 1993, Packer and Rees, 1972). Compared to full restriction, if there is an exchange between compartments, the signal attenuation increases with increasing diffusion time. In practice, an increase in the restriction size has a similar effect as exchange and therefore it is not easy to disentangle them from each other using SDE (Nilsson et al., 2010). For low *b*-values, the SDE signal contains the effect of perfusion (IVIM) (Le Bihan et al., 1988).

Another useful sequence is Double Diffusion Encoding (DDE) which contains two pairs of pulsed-field gradients that are separated from each other with a mixing time *τ* (see Fig. 1b) (Cory et al., 1990, Callaghan, 2011). An alternative realization to this sequence involves overlapping the two pulses in the middle to realize short *τ* values when narrow pulses are not feasible (Özarslan and Basser, 2008). It has been shown that DDE, as well as other multiple encoding schemes (Özarslan and Basser, 2007, Finsterbusch, 2009b, Avram et al., 2013) such as Triple Diffusion Encoding (TDE) (Topgaard, 2017, Ramanna et al., 2020), provide information that is not accessible with single diffusion encoding (Mitra, 1995, Cheng and Cory, 1999, Özarslan and Basser, 2007). This approach has been utilized by several groups for extracting microstructural information (Özarslan et al., 2009b, Jespersen et al., 2013, Benjamini et al., 2014, Ianuş et al., 2016, Yang et al., 2018, Coelho et al., 2019). Varying the relative gradient directions of the two SDE blocks, one is able to estimate microscopic diffusion anisotropy (Cheng and Cory, 1999, Özarslan, 2009, Finsterbusch, 2011, Jespersen et al., 2013, Shemesh, Özarslan et al., 2010a) whereas varying the gradients’ strengths while keeping them orthogonal to each other reveals compartmental kurtosis (Paulsen et al., 2015). In order to estimate exchange, e.g. through the membrane between extra-cellular and intra-cellular spaces, parallel gradients with variable mixing time can be used (Furo and Dvinskikh, 2002, Åslund et al., 2009, Lasič et al., 2011, Sønderby et al., 2012, Nilsson et al., 2013, Ning et al., 2018). In this experiment, the first pair of pulsed-field gradients differentially attenuates the signals in the two compartments, assuming that their diffusivities are different and this gradually returns to equilibrium. To measure exchange, the mixing time is increased gradually and the second block of gradients is used to monitor this equilibrium. Another application of DDE is the estimation of compartment size using parallel and antiparallel gradients with a short mixing time (Koch and Finsterbusch, 2008, Finsterbusch, 2011).

Oscillating diffusion encoding (ODE) can be achieved by changing the single pulsed gradient on either side of the 180° RF pulse to a series of pulsed gradients having the oscillating form (see Fig. 1c) (Callaghan and Stepišnik, 1995). Estimation of the diffusivity in small compartments needs short diffusion times using SDE, this limits the *b*-value that can be achieved and therefore decreases the sensitivity to microscopic features. By repeating multiple pulses in ODE, one can maintain a high *b*-value at short diffusion times. This improves the sensitivity to diffusion coefficients in small pores and therefore the feasibility of estimating small pore sizes (Gore et al., 2010, Xu et al., 2014). The ODE is useful for the estimation of axon diameters in the presence of orientation dispersion because it provides a low signal from free diffusion along the cylinder axis and retains sensitivity to the size (Drobnjak et al., 2016, Nilsson et al., 2017).

**Fig. 2:**
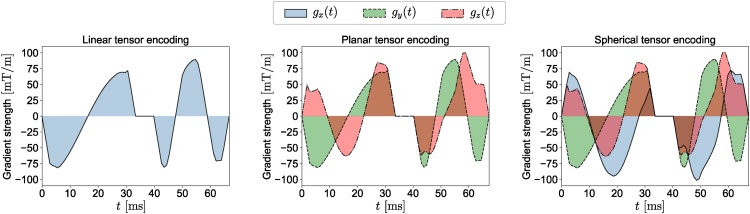
The free gradient waveforms $\text{g}(t)={\left[g_{x}(t),g_{y}(t),g_{z}(t)\right]}^{T}$ of the linear, planar, and spherical tensor encoding. For more details about the gradient waveforms see Section 3.

Although SDE, DDE, and ODE are the most common gradient waveforms there is no reason to limit the shape of the gradient to a rectangular/trapezoidal waveform. Having free gradient waveforms may be more useful than the trapezoidal ones (Drobnjak and Alexander, 2011, Drobnjak et al., 2010). One example is double oscillating diffusion encoding (DODE), which can enhance the estimation of size and shape (Ianuş et al., 2017, Ianuş et al., 2018). Another category is multiple diffusion encoding to disentangle microscopic anisotropy from isotropic diffusion, which is not feasible using SDE alone (Mitra, 1995). A framework called q-space trajectory imaging (QTI) was recently introduced by Westin et al. (2016) to probe tissue using different gradient waveforms. The traditional, pulsed field gradient sequences attempt to probe a point in q-space but in q-space trajectory encoding, time-varying gradients are used to probe a trajectory in q-space. By employing a diffusion tensor distribution model (Jian et al., 2007), the QTI framework provides some microstructural information that is not available in traditional pulsed gradient encoding proposed by Stejskal and Tanner. In multi-dimensional diffusion MRI, the *b*-matrix is defined as an axisymmetric second order tensor (Topgaard, 2017)

(2) $$\text{B}=b(1-b_{\Delta })I_{3}/3+bb_{\Delta }\text{g}{\text{g}}^{T},$$

where ***I***_*3*_ is the identity matrix, **g** is the diffusion gradient direction and the *b*-value, *b*, is defined as the trace of the b-matrix. The eigenvalues of the b-matrix are *b*_∣∣_, $b_{⊥}^{\left(1\right)}$ and $b_{⊥}^{\left(2\right)}$ where $b_{⊥}^{\left(1\right)}=b_{⊥}^{\left(2\right)}=b_{⊥}$ and *b*_∣∣_ is the largest. *b*_Δ_ is defined as *b*_Δ_ = (*b*_∣∣_ − *b*_⊥_)/(*b*_∣∣_ + 2*b*_⊥_). In this framework, SDE and ODE are just special realizations of linear tensor encoding (LTE; Fig. 2) where the *b*-tensor has only one non-zero eigenvalue as all gradients are in the same orientations. DDE is a special case of planar tensor encoding (PTE; Fig. 2) as all gradients line on a plane and the *b*-tensor has two non-zero eigenvalues. In spherical tensor encoding (STE; Fig. 2) the gradients may point in all directions giving rise to a rank-3 b-matrix. Changing *b*_Δ_, we can generate different types of *b*-tensor encoding. For LTE, PTE, and STE, *b*_Δ_ = 1, − 1/2, and 0 respectively (see Topgaard, 2017).

In Table 1 we summarize diffusion encoding schemes aforementioned in this section and provide the information they reveal.

**Table 1:** Summary of diffusion encoding schemes and information they reveal. Each gradient waveform can be incorporated into spin echo or stimulated echo sequences where the latter is preferred for long diffusion times particularly for species in which *T*_2_ is relatively short (e.g., muscle) (Callaghan, 1991).

Encoding	Sequence	Applications	Advantages	Disadvantages	Reference
Single	SDE	Measuring free and restricted diffusion	Easy to implement	Non-optimized in measuring some microstructural features of the tissue	(Stejskal and Tanner, 1965, Huynh et al., 2020, Kaden et al., 2016a)
		Ensemble average propagator			(Kärger and Heink, 1983)
		Diffraction-like features			(Callaghan et al., 1991)
		Modeling anisotropic diffusion, Diffusion tensor imaging			(Basser et al., 1994b)
		Fiber-tract mapping			(Mori et al., 1999)
		Mapping Connectomes			(Sotiropoulos and Zalesky, 2019)
		Neurosurgery, Neuro-oncology			(Panesar et al., 2019, Potgieser et al., 2014)
	ODE	Diffusivity in small compartments, axon diameter			(Callaghan and Stepišnik, 1995)
Double	DDE	Microscopic diffusion anisotropy			(Cory et al., 1990, Cheng and Cory, 1999)
		Compartment size and disambiguation of free and restricted diffusion	Sensitive to microscopic anisotropy	Long acquisition time and long TE compared to SDE	(Mitra, 1995)
		Exchange between different compartments			(Callaghan et al., 2003)
		Compartmental kurtosis			(Paulsen et al., 2015)
	DODE	Enhances the estimation of size and shape			(Ianuş et al., 2017)
Multiple	MDE	Isotropic (spherical tensor) encoding for direct measurement of the trace of the diffusion tensor	Sensitive to microscopic anisotropy	Long acquisition time	(Mori and van Zijl, 1995)
		Same as DDE with potential advantages			(Özarslan and Basser, 2007, Finsterbusch, 2009b, Topgaard, 2017)
Free waveform	QTI	Microscopic and macroscopic diffusion anisotropy, size variance, orientational coherence	Short echo time compared to DDE and MDE	Complicated gradient waveforms, long TE compared to SDE, diffusion time is ill-defined in time domain, needs spectrally matched waveforms for comparison (Lundell et al., 2019)	(Westin et al., 2016)

**Acronyms:** SDE – single diffusion encoding, ODE – oscillating diffusion encoding, DDE – double diffusion encoding, DODE – double oscillating diffusion encoding, MDE – multiple diffusion encoding, QTI – q-space trajectory imaging, TE – echo time.

## 4 Signal representations

Most diffusion MRI based analysis of microstructure falls into two categories: a model of the signal to compute quantitative physical properties of the diffusion and representations of the tissue to acquire tissue-specific metrics. Techniques based on the representation of the signal focus on delineating the diffusion signal attenuation without explicitly considering the underlying tissues that create this attenuation (Basser et al., 1994b, Descoteaux et al., 2011, Jensen et al., 2005, Merlet and Deriche, 2013, Özarslan et al., 2013, Tournier et al., 2011, Ning et al., 2015b, Fick et al., 2016). The most widely used, DT-MRI, employs a tensor to characterize the Gaussian distribution of displacements and MRI signal decay (Basser et al., 1994b, Basser et al., 1994a). DT-MRI (Basser et al., 1994b) has been widely used to determine anisotropy in the tissue *in vivo*. The microstructure of the tissue can be used to determine the effect of aging (Abe et al., 2002), mild traumatic brain injury (Shenton et al., 2012), or some diseases of the central nervous system such as schizophrenia and Alzheimer's disease (Kubicki et al., 2007, Alexander et al., 2019) or even in the preoperative evaluation of tumor grade (Inoue et al., 2005). DT-MRI can provide noninvasive markers of tissue state (Pierpaoli and Basser, 1996) and also can map anatomical connections between different regions of the brain (Mori et al., 1999, Conturo et al., 1999, Basser et al., 2000).

DT-MRI is obtained when the Maclaurin series representation of the natural logarithm of the MR signal is terminated after the first term. Such an expansion is sometimes referred to as the cumulant expansion as the coefficients of different terms correspond to the cumulants of the net displacement distribution (Liu et al., 2004). While the first term, hence the diffusion tensor, is related to the covariance (Basser, 2002), the next term in the series contains the kurtosis of this distribution. Diffusion Kurtosis Imaging (DKI) is obtained when the kurtosis term in addition to the covariance term is preserved in the expansion (Jensen et al., 2005). Doing so extends the validity of the representation towards larger *b*-values. More importantly, a measure of Generalized Kurtosis is provided, which is a 3-dimensional equivalent of the 1-dimensional kurtosis measure used in the DKI literature. However, complex white matter structures such as fiber crossing, bending and branching can obscure the true kurtosis measurements. There are some researches that extend DKI in microstructural environments with orientation heterogeneity (Ankele, 2019, Huynh et al., 2019b, Ankele and Schultz, 2015) and show significantly higher consistency in quantifying microstructure than the conventional DKI in the presence of orientation heterogeneity. Recent works are available on modeling the effects of diffusion in curving structures (Karayumak et al., 2018, Bastiani et al., 2017, Wu et al., 2020, Lee et al., 2020). Diffusion measurements are antipodally symmetric which means the probabilities of displacement along *x* and −*x* are equal, while the distribution of fiber orientations within a voxel is not symmetric in general (Karayumak et al., 2018). Different sub-voxel patterns such as crossing, fanning, and bending, cannot be distinguished if a voxel-wise model is fitted to the signal. Therefore, the spatial information from the neighboring voxels should be considered (Bastiani et al., 2017, Wu et al., 2020)

### 4.1 From q-space to MAP-MRI

The reciprocity of the Fourier Transform (FT) between the ensemble average propagator and q-space (Callaghan et al., 1988) provides another way to characterise diffusion without explicit models. This property is directly used in Diffusion Spectrum Imaging (DSI), which has been employed for mapping complex fiber architectures in tissues by sampling the q-space data in a Cartesian grid and performing a Fourier transform of the measured signal's modulus (Wedeen et al., 2005).

A recently proposed signal-based framework called Mean Apparent Propagator (MAP)-MRI uses a series of basis functions to fit the three-dimensional q-space signal and transform it into diffusion propagators (Özarslan et al., 2013). By efficiently computing the probability density function (PDF) of spin displacements and deriving various metrics from this PDF that accounts for the non-Gaussianity of diffusion, MAP-MRI provides richer information compared to DT-MRI (Avram et al., 2016, Ma et al., 2020).

MAP-MRI represents the diffusion signal *E*(**q**) in 3D q-space and its Fourier transform, mean apparent propagator,

(3) $$P(\text{r})=\int_{{\mathbb{R}}^{3}}E(\text{q})\exp\left(-i2\pi {\text{q}}^{T}\text{r}\right)d\text{q}$$

as a linear combination of some basis functions. For each voxel, a local anatomical reference frame is determined such that the diffusion tensor **D** is diagonalized. Setting

(4) $$\text{A}=2{\text{R}}^{T}\text{D}\text{R}t_{d}=\left[\begin{array}{ccc} u_{x}^{2} & 0 & 0\\ 0 & u_{y}^{2} & 0\\ 0 & 0 & u_{z}^{2} \end{array}\right],$$

where **R**^*T*^ is a rotation matrix that diagonalizes the diffusion tensor and *u*_*x*_, *u*_*y*_, and *u*_*z*_ are scaling factors in the local frame of reference determined by the diffusion time *t*_*d*_ and the eigenvalues *λk*_*k*_ of **D** as $u_{k}^{2}=2\lambda_{k}t_{d}$ (Özarslan et al., 2013). Using a complete set of orthogonal Hermite–Gaussian basis functions, the diffusion signal *E*(**q**) and the propagator *P*(**r**) can be represented as

(5) $$E(\text{q})=\phi^{T}\text{a}\;\overset{\mathrm{FT}}{⟷}\;P(\text{r})=\psi^{T}\text{a},$$

where we use column vector notations **a**(**A**), ***ϕ***(**A**, **q**), and ***ψ***(**A**, **r**) to represent the series coefficients $a_{n_{1}n_{2}n_{3}}$ and corresponding 3D MAP-MRI basis functions in q-space

(6) $$\phi_{n_{1}n_{2}n_{3}}(\text{A},\text{q})=\phi_{n_{1}}(u_{x},q_{x})\phi_{n_{2}}(u_{y},q_{y})\phi_{n_{3}}(u_{z},q_{z})$$

and equivalently in displacement r-space domain

(7) $$\psi_{n_{1}n_{2}n_{3}}(\text{A},\text{r})=\psi_{n_{1}}(u_{x},x)\psi_{n_{2}}(u_{y},y)\psi_{n_{3}}(u_{z},z).$$

The basis functions (6) and (7) are defined by indices *n*_1_, *n*_2_, *n*_3_ with *n*_1_ + *n*_2_ + *n*_3_ = *N* representing the total order in the expansion (truncated at *N*_max_). The relation between dimensional basis functions ϕ_n_(u,q) and *ψ*_*n*_(*u*, *x*) is given by

(8) $$\begin{array}{c} \phi_{n}(u,q)=\frac{i^{-n}}{\sqrt{2^{n}n!}}\exp\left(-\frac{{\left(2\pi \mathrm{q u}\right)}^{2}}{2}\right)H_{n}(2\pi \mathrm{q u})\\ \overset{\mathrm{FT}}{⟷}\;\psi_{n}(u,x)=\frac{1}{\sqrt{2\pi }}\frac{1}{\sqrt{2^{n}n!}u}\exp\left(-\frac{x^{2}}{2u^{2}}\right)H_{n}\left(\frac{x}{u}\right), \end{array}$$

where *H*_*n*_(*x*) is the *n*th order Hermite polynomial. The propagator and diffusion signal are symmetric and real, therefore, there are (*N*_max_ + 2)(*N*_max_ + 4)(2*N*_max_ + 3)/24 coefficients (see Avram et al., 2016). The propagator and diffusion signal can be represented by the same coefficients and different basis functions, which are FT couples. Therefore, it is easy to impose physical constraints like symmetry, non-negativity, and normalization of the propagator when fitting the data (Özarslan et al., 2013, Haije et al., 2020). Analytical descriptors of the propagator can be obtained from MAP-MRI coefficients. For example, return-to-origin probability (RTOP) which is one type of zero-displacement probability (ZDP) can be computed from MAP-MRI coefficients. This index has been suggested as an indicator for restricted diffusion (Wu and Alexander, 2007, Özarslan et al., 2013). Similarly, the deviation from Gaussian diffusion is represented by the non-Gaussianity (NG) index (Özarslan et al., 2013), which is related to the non-Gaussian terms in the MAP-MRI expansion. Rathi et al. (2013) and Ning et al. (2015b) also provided analytical formulations to estimate measures such as RTOP and RTAP. Their method is robust to noise but the number of fibers must be known *a priori*.

**Fig. 3:**
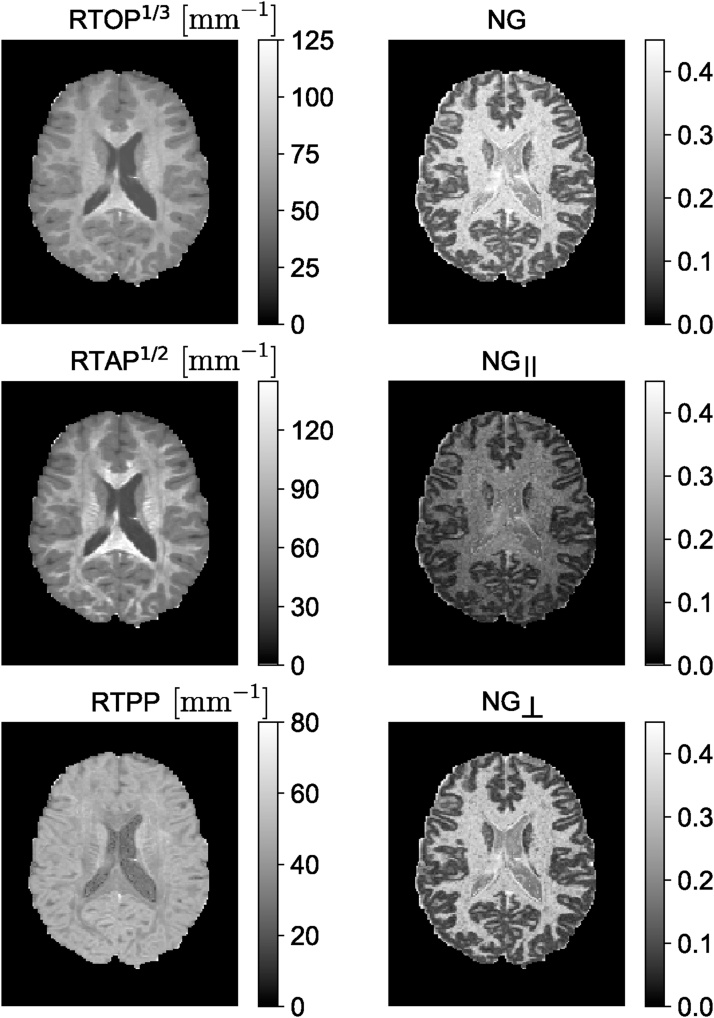
MAP-MRI indices of an HCP WuMinn data with *b* = 1000 , 2000, , and 3000 s/mm^2^. RTOP – return-to-the-origin probability, RTAP – return-to-the-axis probability, RTPP – return-to-the-plane probability, NG – non-Gaussianity.

The MAP basis functions are separable along three dimensions; therefore, the propagator matrices can be decomposed along the axes and planes of the diagonalized diffusion tensor. For example, the presence of restrictive barriers in the radial and axial orientation can be represented by the return-to-axis and return-to-plane probabilities (RTAP and RTPP, respectively). Heterogeneous diffusion in the radial and axial direction is reflected by the parallel and perpendicular non-Gaussianity (NG) indices (NG_∣∣_ and NG_⊥_, respectively). These scalar parameters encode directional information for characterizing diffusion in anisotropic tissues, similar to the diffusivities in the DT-MRI, and could provide WM biomarkers for axonal loss or demyelination. Fig. 3 shows an example of MAP-MRI indices (RTOP, RTAP, RTPP, NG, NG_∣∣_ and NG_⊥_) on a single slice of Human Connectome Project (HCP) data.

### 4.2 Confinement model

DT-MRI is based on the assumption that diffusion is free. There are several ramifications and manifestations of this assumption: (i) DT-MRI does not account for multiple fiber orientations within the voxel; (ii) the signal decay implied by DT-MRI is purely monoexponential, so it does not address the upward curvature of the semi-logarithmic signal vs. *b*-value plots (Pfeuffer et al., 2000); and (iii) the particular dependence of the signal on the timing parameters of the SDE sequence (*δ* and Δ) is substantially different than implied in more realistic scenarios. Indeed, the time-dependence of the MR signal in a homogeneous tissue (Latour et al., 1994) and biophysical studies suggest the presence of restricted diffusion within cells (Beaulieu and Allen, 1994). There are many methods developed over the years to address the first two shortcomings. For example, DT-MRI's limitation in determining fiber crossings has been addressed by using multi-compartment models with different diffusion tensors for each compartment (Inglis et al., 2001, Tuch et al., 2002). However, the third issue has received less attention. The confinement model was developed to address this deficiency (Yolcu et al., 2016).

In the confinement model, the particles diffuse under the influence of a Hookean restoring force (Uhlenbeck and Ornstein, 1930), which constrains the distances the particles can travel. Thus, the diffusion characteristics exhibit features of restricted diffusion. In fact, in several MR studies, the model was employed due to its relative simplicity (Stejskal, 1965, Le Doussal and Sen, 1992, Mitra and Halperin, 1995). Recent work suggests that the confinement model is more than just a simplified approximate model, it is *the effective model of restricted diffusion* under diffusion acquisition scenarios highly relevant to clinical imaging (Özarslan et al., 2017). Its generalization to three-dimensions (Yolcu et al., 2016) is thus a viable alternative to DT-MRI at low diffusion weightings.

The confinement model (Yolcu et al., 2016) is similar to the diffusion tensor representation in spirit. However, taking confinement into account, one can model the time dependence of the diffusion signal which is similar to that for restricted diffusion (Yolcu et al., 2016). The confinement model employs a harmonic confinement instead of direct restricted diffusion, which can encode full anisotropy. For example, the restricted diffusion model of a capped cylinder (Özarslan, 2009) has two length parameters due to its transverse isotropy while the confinement model has three distinct size parameters just like the diffusion tensor model. Moreover, it is possible, though sometimes tedious, to obtain analytical expressions of the signal intensity for general gradient waveforms. As an example, the normalized signal for the SDE experiments is given by

(9) $$S=\exp(-{\text{G}}^{T}\text{A}\text{G})\;$$

with

(10) $$\begin{array}{c} \text{A}=-D\gamma^{2}\Omega^{-3}[\left(1-\exp(-\Omega \Delta )\right){\left(1-\exp(-\Omega \delta )\right)}^{2}\exp(\Omega \delta )\\ \begin{array}{c} \begin{array}{c} -(1-\exp(-2\Omega \delta ))\exp(\Omega \delta )+2\Omega \delta \end{array} \end{array}]. \end{array}$$

Here **Ω** = *βD***f**, where *β* = (*k*_*B*_*T*)^−1^ with *k*_*B*_ the Boltzmann constant and *T* the temperature, **f** is the tensorial spring constant, *D* is the diffusion coefficient, and **G** is the magnetic field gradient vector. It should be noted that *D* is the bulk diffusivity, hence it is not affected by the characteristics of the restricting geometry. Thus, when the stiffness tensor goes to 0, one obtains the expression given by Eq. (1) as expected.

**Fig. 4:**
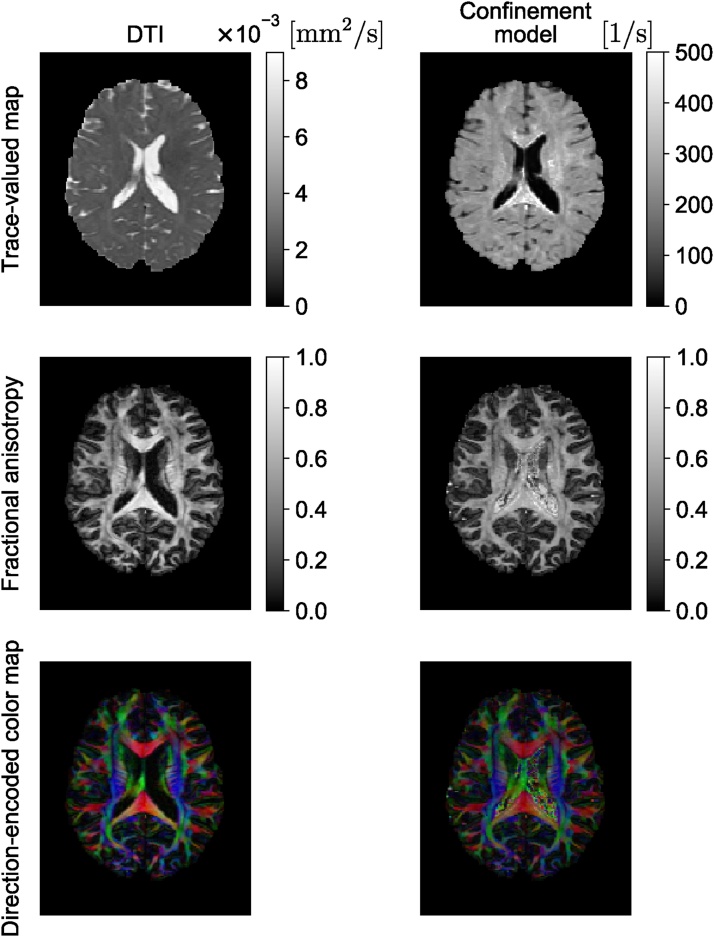
Comparison of quantitative measures derived from DT-MRI and confinement models when applied to the signal from the whole voxel (see Section 4.2). For the new model, the direction-encoded color map was computed by color-coding the direction of the eigenvector of the stiffness tensor associated with its *smallest* eigenvalue. In the color-coded map, red, green, and blue represent fibers running along the right-left, anterior-posterior, and superior-inferior axes, respectively.

The confinement model is ideally suited to representing the signal for each restricted subdomain of a heterogeneous medium (Yolcu et al., 2016, Yolcu et al., 2019) in a multi-compartment model (see Section 5.1). However, it was also employed to represent the signal from the whole voxel in a way similar to how DT-MRI is employed. Afzali et al. (2015) have shown the feasibility of this model on in vivo brain images while (Zucchelli et al., 2016) have reported improved performance when applied on data with varied timing parameters. Fig. 4 shows the comparison between the diffusion tensor and confinement tensor indices as an example on a single slice of Human Connectome Project (HCP) data (McNab et al., 2013). In Fig. 4, we illustrate the trace, FA, and direction-encoded color (DEC) maps for DT-MRI (left) and the confinement (right) models. In general, one expects a negative image in trace(**A**) maps as regions with large diffusivity should correspond to springs with small stiffness values. Such behavior is indeed observed in the trace maps. The most visible difference is the presence of hyperintense regions in the trace(**A**) map scattered within the white matter areas. The FA maps contain the same information for the most part. The DEC maps are also similar when the eigenvector corresponding to the smallest eigenvalue of **A** is used. The FA map from the confinement model seems to be noisy, especially in the ventricle. The structure in this region is simpler and more homogeneous than other regions, thus, a noisy FA map is not expected. The apparently noisy and anisotropic outcome in the ventricles is fully explained by the limited sensitivity of the signal on the stiffness values when these values are small.

### 4.3 Power-law scaling of the diffusion signal

Water molecules in the intra- and extra-cellular spaces form two compartments with different behaviors (Assaf et al., 2004). The intracellular compartment is assumed as thin cylinders. Neurites have a very small diameter and one can neglect the effect of the perpendicular diffusivity on the data acquired on a clinical scanner. This behaviour (Behrens et al., 2003) was reported for water and for N-acetyl-L-aspartate (NAA) diffusion (Kroenke et al., 2004).

The power-law behavior in diffusion MRI was first observed by Köpf et al. (1996) who conducted experiments on nonneural tissues exploiting the fringe field of an MR scanner, which features extremely large gradients. They reported fractional values for the exponent characterizing the tail of the MR signal decay and those values varied from specimen to specimen; these observations were interpreted within the framework of fractional Brownian dynamics and an analysis involving fractal concepts was performed (Köpf et al., 1998). Yablonskiy et al. (2003) introduced a statistical model employing a distribution of diffusion coefficients, and predicted a decay characterized by *b*^−1^. Jian et al. (2007) proposed a tensor distribution model for the diffusion-weighted MR signal and demonstrated that their model could provide any non-negative integer and half-integer exponent depending on the characteristics of the tensor distribution. They adopted a decay rate *b*^−2^ in their implementation, which is consistent with the Debye–Porod law adapted to the field of diffusion MR (Sen et al., 1995), and successfully resolved the orientational complexity of the tissue within each voxel.

When the problem does not involve resolving fiber orientations, one can opt to suppress the anisotropy of the detected signal; doing so reduces the multi-dimensional signal profile into a lower-dimensional one that depends only on the microstructural features. Earlier studies on DDE proposed such a reduction. For example, Özarslan and Basser (2008) employed an irreducible representation of the orientation distribution function and taking its “isotropic component” to rid the signal of the effects of ensemble anisotropy. Jespersen et al. (2013) achieved the same effect by employing numerical integration of the signal profile. In the case of SDE, MAP-MRI has introduced the propagator anisotropy (PA) index (Özarslan et al., 2013), which is based on the dissimilarity of the isotropically-averaged signal from the actual (anisotropic) one. Kaden et al. (2016b) employ a simple arithmetic averaging over each shell in multi-shell data to obtain the one-dimensional signal vs. *b*-value profile while (Szczepankiewicz et al., 2017) propose a weighted-averaging scheme for this purpose.

Using any of the above-mentioned methods, one obtains a representation of the so-called powder-(or direction- or orientationally-)averaged signal, which is the same signal for a specimen that has undergone “powdering”—a process employed for analyzing solid-state samples that involves grinding the specimen to eliminate any orientational coherence in its structure. Such specimens have been studied via MR before (Callaghan et al., 1979, Edén, 2003). It was reported recently that diffusion MR images after direction-averaging have good contrast between GM, WM, and CSF (Cheng et al., 2020).

For the powder-averaged signal, the diffusion attenuation is a function of the orientation-invariant aspects of the diffusion process as well as the experimental scheme employed for encoding diffusion. For example, Eriksson et al. (2015) provide the expression for a specimen consisting of identical, though possibly incoherently-aligned, collection of subdomains, given by

(11) $$\begin{array}{c} \bar{S}(b)=\frac{\sqrt{\pi }\exp\left(-\frac{b}{3}\left(D^{||}+2D^{⊥}-b_{\Delta }(D^{||}-D^{⊥})\right)\right)}{2\sqrt{bb_{\Delta }(D^{||}-D^{⊥})}}\mathrm{erf}\left(\sqrt{bb_{\Delta }(D^{||}-D^{⊥})}\right) \end{array},$$

where $\bar{S}$ is the normalized orientationally-averaged signal and *D*^∣∣^ and *D*^⊥^ are the parallel and perpendicular diffusivities, respectively. Here, the measurement tensor is also axisymmetric.

The above expression suggests that if the diffusivity in the perpendicular direction is zero, the orientationally-averaged SDE signal obeys a power-law $\bar{S}=\beta b^{-\alpha }$ with *α* = −1/2. Therefore, the presence of this particular power-law would suggest vanishing diffusivity in the directions perpendicular to the fiber direction, justifying the “stick” model of neurites.^1^ The observation of this particular power-law decay was recently reported for white matter by McKinnon et al. (2017) and Veraart et al. (2019).

Özarslan et al. (2018) pointed out that such slow decay with exponent *α* = −1/2 could only occur in an intermediate range of *b*-values as the true asymptotic behavior of the powder-averaged signal is governed by a power law with *α* = 2 for narrow pulses (due to Debye–Porod law; Sen et al., 1995), and faster than any power law for longer pulses (Özarslan et al., 2018). Recently, Veraart et al. (2020) exploited such deviation of the SDE signal decay curve from the $\bar{S}=\beta b^{-1/2}$ behaviour at large *b*-values to estimate the diameter of the axons in white matter.

Herberthson et al. (2019) studied the effect of *b*-tensor shape on the diffusion-weighted signal at high *b*-values and generalized (11) to the cases involving non-axisymmetric diffusion and/or *b*-tensors. They predicted another power-law with *α* = 1 when one of these tensors is of rank 1 and the other is of rank 2. Afzali et al. (2020) showed the power-law relationship between PTE diffusion MR signal and the *b*-value. They observed the exponent *α* = 1 using planar tensor encoding *in vivo*.

Yolcu et al. (2019) considered powder-averaged SDE and DDE measurements and derived exact expressions for the signal when the compartment is defined by a confinement tensor (Yolcu et al., 2016). They predict that for confined diffusion within stick-like geometries, the same kind of power-laws persist while the coefficient *β* exhibits a different dependence on the timing parameters of the sequence if diffusion along the stick is confined.

In gray matter, McKinnon et al. (2017) and Afzali et al. (2020) observed an exponent (*α*) larger than in white matter using linear and planar tensor encoding, respectively. Three proposals have been made to explain such different behaviour in gray-matter; one is the permeability of the cell membrane resulting in a significant exchange between the intra and extra-cellular spaces (McKinnon et al., 2017). Another one is the curvature of the neural projections (Özarslan et al., 2018), and the third one is the abundance of a three-dimensional compartment (e.g. spherical), which could be due to the cell bodies (Palombo et al., 2018b).

The effect of diffusion in curving structures on the MR signal has been investigated in different contexts (Özarslan et al., 2009a, Jespersen and Buhl, 2011, Nilsson et al., 2012, Reisert et al., 2012, Pizzolato et al., 2015, Cetin Karayumak et al., 2018). Recently, Özarslan et al. (2018) studied the effect of size and curvature of the neurites and glial projections in the context of the power-laws. They showed that for one-dimensional diffusion along curvy structures, longer pulse durations lead to a decay steeper than *b*^−1/2^ while the power-law with *α* = 1/2 persists when the gradient pulses are narrow. Therefore, the curvature effect may be a significant contributing factor to the steeper attenuation observed in clinical scanners due to the long pulse durations employed.

**Fig. 5:**
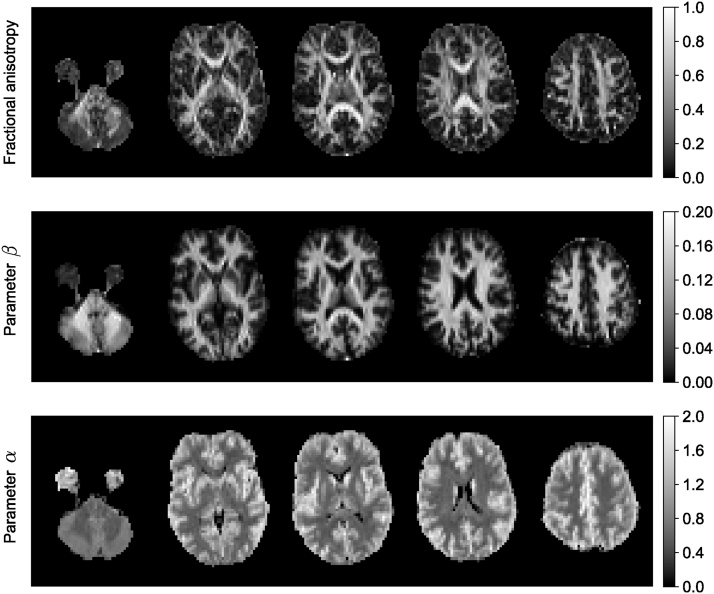
Estimated fractional anisotropy, and the results of power-law fit (*S*/*S*(0) = *βb*^−*α*^) to the brain image.

Fig. 5 shows the maps of estimated fractional anisotropy (FA), *β* and *α* (*S*/*S*(0) = *βb*^−*α*^) values for five different slices of a brain image. The data for this experiment were acquired with 61 gradient directions per shell using LTE on a 3T Connectom MR imaging system (Siemens Healthineers, Erlangen, Germany). The voxel size was 3 mm isotropic, TE = 88 ms, TR = 3000 ms, *b* = 6000, 7500, 9000, 10,500 s/mm^2^. The estimated β map has a similar appearance to the FA map, on the whole. However, notably in regions with known fibre crossings (e.g. near the horns of the lateral ventricles), the FA has the well-known ‘dip,’ while the β-map is more homogeneous. The exponent *α* is very small in CSF because no signal remains from free diffusion at high *b*-values. The decay in gray matter is faster than white matter as discussed above.

## 5 Microstructure models

This section explains methods that relate the diffusion signal to the features of the brain microstructure and discusses some of the applications in biomedical sciences.

### 5.1 Multi-compartment models

Many microstructure models developed over the years for interpreting the diffusion MR data employ a multi-compartmental approach wherein the signal is written as the sum of contributions from different structures making up the neural tissue (Stanisz et al., 1997; Alexander, 2008; Alexander et al., 2010; Kaden et al., 2016b; Panagiotaki et al., 2012; Scherrer et al., 2016).

Utilizing biophysical models in diffusion MRI to estimate the microstructure of the underlying tissue resembles the physical chemistry field where these types of models were used to determine the microstructure of the sample (Schmidt-Rohr and Spiess, 2012). The size distribution of oil droplets was quantified by a model of spheres with log-normal distributed radii (Packer and Rees, 1972). After DT-MRI was proposed (Basser et al., 1994b), the eigenvalues of the diffusion tensor or related indices such as mean diffusivity (MD) or fractional anisotropy (FA) were interpreted as measures of fiber density or myelination (Brubaker et al., 2009). In brain regions containing highly parallel fibers such as CC, these parameters may be able to reflect such tissue properties (Stikov et al., 2011). But in general, because of the fibres’ orientational dispersion (De Santis et al., 2014), simple indices such as FA and MD cannot provide proper information about the fiber density and more complicated models are necessary to disentangle the effect of dispersion from the fiber density (Zhang et al., 2012).

In this section, we focus on models that consider the signal in each voxel as the sum of several compartments each of which could represent a single cellular compartment. This method tries to find compartment-specific properties and is different from signal models such as DT-MRI (Basser et al., 1994a), DKI (Jensen et al., 2005), q-space imaging (Callaghan, 1993; King et al., 1994; Wu et al., 2008), DSI (Wedeen et al., 2005) and MAP-MRI (Özarslan et al., 2013) that attempt to characterize voxel-averaged quantities. The biophysical models relate the signal directly to the microstructural features of the tissue. Similar to representations, the parameters of the model can be estimated by fitting the model to the signal. For example, the axons can be modeled as cylinders, and fitting the signal expression for diffusion inside cylinders to the collected data could reveal the diameter of the axons.

Stanisz et al. (1997) were the first to use a multi-compartment model to study the nerve-tissue microstructure. They considered separate compartments for glial cells, axons, and extracellular space and tried to estimate the signal fraction of each compartment and the size of cells. The glial cells are modeled as spheres and axons as ellipsoids where restricted diffusion is defined by their geometry. Diffusion in the extracellular space is approximated with a tortuosity model. Tortuosity refers to the reduction in apparent diffusivity compared to the bulk diffusivity in an environment with obstacles (Fried and Combarnous, 1971, Gray, 1975, Lehner, 1979; Nicholson and Phillips, 1981). The particle mobility is determined by this factor (Nicholson and Phillips, 1981). Stanisz et al. (1997) used the method presented in Szafer et al. (1995) that relates the packing density to a reduction in diffusivity as a function of the signal fraction of the obstacle; higher signal fractions lead to lower extracellular diffusivity. Their model also considers exchange between intracellular and extracellular compartments using Kärger's model (Kärger et al., 1988).

Recent models of WM represent axons as straight, impermeable cylinders. The ball and stick model (Behrens et al., 2003) considers the axons as sticks (cylinders with zero diameters) with a zero perpendicular diffusivity and the extracellular part is modeled as isotropic diffusion (ball). The model assumes the same values for extra- and intra-cellular diffusivity. The next model in the evolution of microstructural mapping was proposed by Assaf and Basser (2005), Assaf et al. (2004) composite hindered and restricted models of diffusion (CHARMED) where the distribution of the cylinder radii is assumed as gamma distribution (Assaf et al., 2008). In this model, the intra-axonal space is modeled using cylinders with parallel and perpendicular diffusivities. The extracellular compartment is modeled by a diffusion tensor without any tortuosity constraint. In Kroenke et al. (2004), Jespersen et al. (2007), Fieremans et al. (2011), Hui et al. (2015) the model is simplified by considering a stick model for axons. In Barazany et al. (2009) a free water compartment is added to the model to consider the CSF. The AxCaliber model (Barazany et al., 2009, Assaf et al., 2008) is based on CHARMED and is used to estimate the axon diameter distribution (ADD), which requires knowledge about the fiber direction. More recent implementations of AxCaliber use a continuous Poisson rather than a Gamma distribution, which reduces the number of fitted parameters (De Santis et al., 2016). ActiveAx (Alexander et al., 2010, Alexander, 2008) simplifies and combines the assumptions in Stanisz's model (Stanisz et al., 1997) and the CHARMED model (Assaf and Basser, 2005) to produce the minimal model of white matter diffusion (MMWMD) (Dyrby et al., 2013). The simplifications are, considering one axon radius, a fixed diffusivity for intra- and extra-axonal compartments and considering a tortuosity constraint for the extra-cellular compartment (Szafer et al., 1995). The MMWMD has an isotropic restricted compartment similar to the glial cell model in Stanisz et al. (1997). Further studies, such as Panagiotaki et al. (2012) and Ferizi et al. (2017) made a taxonomy of compartment models of WM.

An intra-axonal compartment assumed in MMWMD does not consider the bending and fanning fibers. Spherical deconvolution (Tournier et al., 2004; Kaden et al., 2007; Anderson, 2005) aims to estimate the fiber orientation distribution. This technique does not consider the microstructure of the tissue. The fiber crossing and dispersion (Behrens et al., 2007; Sotiropoulos et al., 2012; Zhang et al., 2011) can be considered in ball-stick, MMWMD, AxCaliber3D models (Barazany et al., 2011). Models of complex orientation distribution can be used both in gray matter and white matter. Jespersen et al. (2007) first explored this by a two-compartment model of neurites. They consider the spherical harmonic representation of orientation distribution function. They also considered a perpendicular diffusivity to reflect the effect of radius, bending, undulation, and exchange. The extra-axonal compartment is modeled with a diffusion tensor.

The next model in the evolution of microstructural mapping was a simpler model proposed for neurite orientation dispersion and density imaging (NODDI) (Zhang et al., 2012). NODDI is a simplified version of MMWMD (Zhang et al., 2011). The orientation distribution function in the NODDI model is assumed as a Watson distribution. Having all these assumptions makes the fitting stable but the estimated parameters may be biased (Lampinen et al., 2018; Jelescu et al., 2016). Other models tried to relax these constraints and estimate the parameters instead of fixing them. Tariq et al. (2016) used a Bingham instead of Watson distribution. Fiber crossing is considered in Farooq et al. (2016). Kaden et al. (2016a) used a spherical mean technique that does not need any assumption about the orientation distribution of the fibers and it allows the estimation of the diffusivities which was fixed in NODDI model. Jelescu et al. (2016) extended the two-compartment model by releasing all the constraints on intra- and extra-cellular diffusivities and they have shown that there is a degeneracy in the fitting of the parameters in this two-compartment model using conventional diffusion imaging. The problem with these models is that the fitting is not stable anymore and different sets of parameters lead to the same solution. Different strategies were proposed to solve the problem of degeneracy in the fitting of the model parameters. Veraart et al. (2018) proposed to use echo time (TEDDI) as an extra measurement to solve the degeneracy problem. The problem with this model is that it adds two more parameters which makes the fitting more complicated. Fieremans et al. (2018) suggested using a combination of linear and spherical tensor encoding to improve the estimation of some of the parameters of the model. Reisert et al. (2018) and Coelho et al. (2019) used analytical solutions to show that the combination of linear and planar tensor encoding solves this problem. They have also shown that the spherical tensor encoding does not help to solve the degeneracy in the estimation of parameters in this two-compartment model. Lampinen et al. (2017) show that adding the spherical tensor encoding acquisition helps to solve this degeneracy problem. A framework for machine learning, reconstruction, optimization, and microstructure modeling called MicroLearn is provided by Fadnavis et al., 2019b, Fadnavis et al., 2019a which is part of Diffusion Imaging In Python (DIPY) library (Garyfallidis et al., 2014).

There is another category of the compartment models that focuses on the statistical modeling of the tissue heterogeneity. One of these techniques is diffusion basis spectrum imaging (DBSI) (Wang et al., 2011). It models the extra-axonal space as a spectrum of isotropic diffusion tensors. This spectrum is defined by a function that determines the fraction of isotropic tensors with a specific diffusivity. A similar idea exists in the generalization of the ball and stick model (Jbabdi et al., 2012) that assumes a spectrum of diffusivities with gamma distribution. Restricted spectrum imaging (White et al., 2013) considers diffusivity spectra for both intra- and extra-axonal diffusivities. Recently, Scherrer et al. (2016) have proposed a model to capture heterogeneity from restricted, hindered and isotropic diffusion modeling heterogeneity by a gamma distribution (Jian et al., 2007; Jbabdi et al., 2012; Ramirez-Manzanares et al., 2007; Leow et al., 2009). Ning et al. (2017a) proposed a method which connects time-varying diffusion and spatially varying diffusivity. This is done without assuming the number of compartments in the model, and it allows determination of the level of disturbance that is caused by the complexity of the medium. A comparison of different power-laws (in the time domain) (Özarslan et al., 2006; Novikov et al., 2014) is reported in the work by Ning et al. (2017b). There are different methods to determine the compartment size distribution. Packer and Rees (1972) assumed a log-normal distribution for the compartment size and used the molecular displacement measurements to estimate the parameters of the distribution. Özarslan et al. (2011b) proposed a strategy to measure all moments of the compartment size distribution directly from the diffusion signal decay. This method does not have the assumption of known parametric size distribution. However, this is accomplished in “ideal” experiments involving narrow pulses and long diffusion times. Also, the q-value sampling has to be dense and broad to provide significant signal attenuation. Although obtaining the actual size distribution is prohibitively difficult, experiments conducted on well-characterized phantoms demonstrated that a contrast based on the moments of the distribution of cylinder radii can be obtained using this method (Özarslan et al., 2011b).

In Table 2 we summarize different multi-compartment models along with assumptions behind them, acquisition schemes and parameters of interest of each technique.

**Table 2:** Summary of different multi-compartment models.

Parameter of interest	Recommended methods	Assumptions	Acquisition	Reference
Axon diameter	CHARMED	Single diameter, no exchange between intra- and extracellular compartments, axons are assumed as straight cylinders	PGSE with variable diffusion time	(Assaf and Basser, 2005, Assaf et al., 2004)
	AxCaliber	Gamma distribution for axon diameters	PGSE	(Assaf et al., 2008, Barazany et al., 2009)
	ActiveAx	Single diameter	PGSE	(Alexander et al., 2010)
	MMWMD	Single diameter	PGSE	(Dyrby et al., 2013)
	ActiveAx-D	Axon diameter index, dictionary-based fitting	PGSE	(Sepehrband et al., 2016b)
	Axon diameter mapping	Watson ODF, single diameter	PGSE	(Zhang et al., 2011)
Diffusivity and signal fraction	Ball + Stick	Same intra and extracellular diffusivity	PGSE, clinical	(Behrens et al., 2003)
	Stick + Tensor	Different intra- and extracellular diffusivity	PGSE	(Kroenke et al., 2004, Jespersen et al., 2007, Fieremans et al., 2011, Hui et al., 2015)
	NODDI	Watson ODF, fixing intracellular diffusivities, tortuosity constraint	PGSE, clinical	(Zhang et al., 2012)
	NODDIDA	Watson ODF, variable diffusivities, no tortuosity constraint	PGSE	(Jelescu et al., 2016)
	TEDDI	Variable TE	PGSE	(Veraart et al., 2018)
	Standard Model	Variable diffusivities, no tortuosity constraint	PGSE	(Novikov et al., 2018)
	LEMONADE	Rotation invariant mapping	PGSE	(Novikov et al., 2016)
	LEMONADE(t)	Time dependency is considered	PGSE	(Lee et al., 2018a)
			B-tensor encoding	(Fieremans et al., 2018, Reisert et al., 2018, Coelho et al., 2019, Lampinen et al., 2017, Lampinen et al., 2018, Lampinen et al., 2020)
Sphere size, diffusivity and signal fraction	Ball + Stick + sphere	No exchange	PGSE	(Palombo et al., 2020)

**Acronyms:** CHARMED – composite hindered and restricted model of diffusion, PGSE – pulsed gradient spin echo, MMWMD – minimal model of white matter diffusion, ODF – orientation distribution function, NODDI – neurite orientation dispersion and density imaging, NODDIDA – NODDI with diffusivity assessment, TEDDI – TE dependent diffusion imaging, TE – echo time, LEMONADE – linearly estimated moments provide orientations of neurites and their diffusivities exactly.

### 5.2 Curvedness of neural trajectories and estimating the axon diameter

In Section 4.3, we discussed the effect of neurite curvature on the power-law scaling of the diffusion MR signal. Here, we consider its effect on the axonal diameter estimations.

Most methods extract the size of axons using the effect of time-dependent diffusion. This strategy works if the axons are straight. The curvedness of the axonal trajectory will affect signal decay and the estimated size (Brabec et al., 2019). Besides, the microscopic orientation dispersion will affect the estimated size. The estimated diameter with the straight-cylinder assumption is dependent on the microscopic orientation dispersion and the undulation amplitude. The undulation can be represented in the power-law behavior of the signal. If we consider this in our experiments we can separate the undulating fibers from the straight ones.

Modeling axons as straight impermeable cylinders is widely used in diffusion MRI studies. However, the validity of this assumption is not yet proven (Lee et al., 2018b; Nilsson et al., 2017). For example, the diameter of an axon may vary along its length (Lee et al., 2018b; Perge et al., 2009). Besides, axons may have fine morphological features such as spines, leaflets, or beads (Palombo et al., 2018a). Maybe the most important aspect among the all mentioned above is that axons are not straight (Nilsson et al., 2012). Some axons have sinusoidal trajectories with an undulation amplitude in the order of magnitude higher than the axon diameter. Such axons are present extracranially such as the phrenic nerve (Lontis et al., 2008) and in the cranial nerves such as the roots of the trigeminal nerve (Kaplan, 1960). Also, the undulation is present in some parts of the central nervous system such as corona radiata, optical nerve radiations, and the corpus callosum (Nilsson et al., 2012, Williams, 1995). Considering the undulation effect is important because it may result in the overestimation of the axonal diameter (Nilsson et al., 2012). (Brabec et al., 2019) investigated the features of non-straight axons that can be captured by dMRI and also explored how these features could complicate an analysis based on the straight cylinder assumption. The time-dependence effect observed in white matter is subtle and may come from the undulation instead of axonal diameter (Nilsson et al., 2009). Axonal diameter indices of 3–12 microns were reported using the ActiveAx model to the corpus callosum in the human and monkey brain (Alexander et al., 2010). The presence of a weak undulation of axons with a diameter below 3–5 microns, which is biologically feasible shows similar results (Nilsson et al., 2017). The reason that undulation is misinterpreted as the axonal diameter is that the undulating thin-fibers (Here ‘thin-fiber’ is functionally equivalent to a ‘stick’, i.e., the diffusivity perpendicular to the local long-axis of the fibre is zero) and straight cylinders have the same diffusion behavior in the regions that are sensitized by common diffusion encoding protocols. Nilsson et al. (2012) studied the effect of undulation on the diffusion propagator and they showed that the width of the propagator reflects the undulation amplitude instead of the cylinder diameter (Nilsson et al., 2012).

The undulating thin-fiber model is only valid for the small-diameter axons (smaller than 4–5 microns) in the brain white matter, which is the same as the resolution limit in clinical scanners (Nilsson et al., 2017). Below this limit, axons can be represented by thin-fibers. Note that large axons exist to a limited extent in the brain but they are more common in the spine and the nerves outside the central nervous system. Small axons are present in the corpus callosum (Aboitiz et al., 1992; Liewald et al., 2014; Mikula et al., 2012), optic nerve (Jonas et al., 1990) or phrenic nerve (Takagi et al., 2009).

### 5.3 Limitations of multi-compartment models

Available models have several limitations. The main feature of multi-compartment models is that they divide the signal into separate compartments. It is necessary to disentangle the signal into several components but it is difficult to assess the validity of the result. Although the previous studies show that WM is composed of intra- and extra-cellular compartments, the presence of distinguishable compartments for glial cells and CSF has not been shown explicitly. The signal fraction estimated from the current models is weighted by *T*_1_ and *T*_2_ relaxation times. The straight model for axons is clearly an oversimplification for the vast majority of axons in the brain. In the axons, there is undulation (Nilsson et al., 2012) and dendritic branching (Palombo et al., 2016). The impermeability assumption (in the slow-exchange domain) can be valid for healthy WM but it can be violated in pathology (Åslund et al., 2009; Lasič et al., 2011; Nedjati-Gilani et al., 2017). Extra-axonal space is assumed to exhibit time-independent diffusion but some experiments (Silva et al., 2002; Shemesh et al., 2011) suggest that in a densely packed environment, the extracellular component may show some time-dependency. In other experiments (Burcaw et al., 2015; De Santis et al., 2016) the time dependence of the extracellular component is not negligible when the diffusion time is about 10–100 ms. Some models consider fixed or constrained diffusivity while some recent experiments show that this assumption is not valid in the brain (Lampinen et al., 2017; Jelescu et al., 2016; Kaden et al., 2016b; Hutchinson et al., 2017).

Models usually consider biophysical influences on the signal. However, using the available practical acquisitions, a small set of parameters can be estimated. Some constraints such as fixing parameters, ignoring some effects (Jelescu et al., 2016), enforcing the relationship between the model parameters, or imposing prior distribution may bias the estimation of the remaining parameters.

With multi-compartment models, one often uses “volume fraction” to describe a compartment, when in fact the fraction computed is “signal fraction”. The concept of ‘signal fraction’ is more appropriate to describe what has always been referred to as ‘volume fraction’ (Frigo et al., 2020). These two concepts are not interchangeable. ‘Volume fraction’ measures the volume of the tissue compartment that is present in the voxel. To better understand this difference, consider given proton density [*H*], the repetition time TR, the echo time TE, and *T*_1_ and *T*_2_ of a tissue, the signal intensity in a spin-echo experiment is *S* ∼ [*H*](1 − exp(− TR/*T*_1_)) exp(− TE/*T*_2_). The *T*_2_ time of the CSF is smaller than the white matter, therefore, the signal amplitude in the CSF is larger. Simple normalization of dMRI signal by the non-diffusion-weighted signal *S*(0) does not take into account this difference and assumes that different tissues have the same *S*(0) response which is not true (Just and Thelen, 1988). This issue cannot be solved by acquiring images with multiple TE ((Veraart et al., 2018)) because this method provides the estimates of the composite *T*_2_ in each voxel and the *T*_2_ of the single compartment is still unknown. All the multi-compartment models that try to estimate the volume fraction of tissue are actually describing the signal fraction.

### 5.4 The effect of acquisition method on the parameter estimation

The choice of experimental design affects the parameter estimates in any model-based estimation technique. Optimal experimental design means the right choice of the pulse sequence and the acquisition parameters to maximise sensitivity to the parameters of the model (Koay et al., 2012). In diffusion MRI, the acquisition parameters to consider might, depending on the complexity of the model, range from simply having to consider the *b*-value, to having to consider a whole range of parameters including *δ*, Δ, **g**, TE, etc. The optimal design will also maximise the SNR per unit time as the acquisition time in *in vivo* studies is usually limited by participant's compliance. For *in vivo* studies, the acquisition time has to stay in a reasonable range.

### 5.5 Effect of model fitting

After deciding the choice of model and acquiring the data, we have to fit the model to the data. The standard method is to use maximum likelihood estimations via non-linear fitting such as gradient descent in each voxel separately. In the fitting, a best-guess parameter estimate is reported. Also, the gradient descent techniques usually provide an additional measure of confidence in the parameter estimate. Sampling methods such as Markov chain Monte Carlo (MCMC) sample the posterior distribution on the parameter values and can provide a confidence interval in each parameter estimate and can avoid the local minima problem which is common in gradient descent.

Recently, several linear fitting approaches have been reported in the diffusion MRI modeling literature, including convex optimisation and dictionary-based techniques. Linear approaches avoid the local minima and are faster than the non-linear methods, but reduce the precision of the final estimates. Methods such as AMICO (Daducci et al., 2015), LEMONADE (Novikov et al., 2015), WMTI (Fieremans et al., 2011, Sepehrband et al., 2016b) are examples of this linearization. However, finding the confidence interval from these types of techniques is not straightforward. Haije et al. (2020) investigated the necessity of non-negativity constraints for diffusion MRI models and Harms et al. (2017) proposed a fast and robust optimization method for diffusion MRI microstructure models.

Considering local inter-voxel coherence of tissue properties sometimes improves the results, because instead of treating each voxel independent from the neighbors, we can analyze the voxels with similar signal decay. In WM, the macroscopic continuity (Sherbondy et al., 2010) of the fibers provides more constraints on the parameter estimates. Morgan (2012) used this dependency to fit the trend in axon diameter across the CC. Scherrer et al. (2016) used the BOBYQA algorithm (Powell, 2009) to improve the fitting of the DIAMOND model. The recent combination of global tractography and microstructure is also available (Reisert et al., 2014; Sherbondy et al., 2010).

Besides conventional model-fitting methods, deep-learning-based methods have recently gained attention (Ye, 2017; Ye et al., 2019). Deep learning approaches have some advantages over conventional fitting methods; Conventional methods can be very time consuming while deep learning methods can be very fast once the training procedure has been completed. Spatial consistency of diffusion signals can be used to reduce the effect of noise in deep learning methods while in conventional fitting methods noise is a serious issue. It can handle a large amount of data while this is not easy in conventional fitting approaches. A lot of deep learning methods can handle highly nonlinear relationships that cannot be handled using a normal fitting approach. There are some disadvantages in using deep learning methods comparing to the conventional fitting methods; Deep learning approaches usually need a large data set to train, computationally very expensive, requiring a large amount of memory and computational resources, and usually require very advanced optimization techniques.

### 5.6 Validation

One important aspect of developing microstructural models is validation. Most, if not all, models establish validity with numerical experiments based on some simulated models or hypothetical assumptions about tissue architecture. Here we discuss different validation techniques and their advantages and disadvantages.

A good microstructural model should be able to capture the underlying features of the tissue. To evaluate the performance of a model, different strategies can be used including simulations and a combination of dMRI and microscopy measurement in tissue and phantom. Numerical simulations usually provide high control while it is far from the real data, while the microscopy measurements are from the real data and controlling different factors in the measurement is not as straightforward as in the numerical simulations.

In Table 3 we briefly summarize evaluation techniques presented in this section along with advantages and disadvantages of each technique.

**Table 3:** Summary of evaluation techniques.

Method	Advantages	Disadvantages
Numerical analysis	Different factors are under control	Simulated data is generated from the model
Monte Carlo	Different factors are under control. Complex substrates can be modeled	Data does not come from measurements
Phantoms	Ground-truth values are controlled	Measured data is similar to ideal samples
Fixed tissue	Ground-truth values are not controlled	The time between death and fixation should be short. The fixation process may change the microstructure
In vivo + ex vivo	Direct validation for tumour cells	Impossible in healthy human

#### 5.6.1 Numerical analysis

To investigate the robustness of parameter estimates under ideal conditions or controlled noisy situations, numerical simulations can be used. The basic idea in these types of simulations is to generate the signal for a given measurement protocol, add different levels of noise, and fit the model. By simulating and fitting the same model, one can establish the effects of noise level and measurement protocol on the parameter estimates (Jones and Basser, 2004). In addition, the resolution limit and the range in which parameters can be estimated with high accuracy (Nilsson et al., 2010; Alexander et al., 2001) can be provided at this stage. Numerical simulations also provide the interplay between parameter estimates and hardware constraints. For example, the maximum gradient strength of the scanner affects the resolution limit of the axon diameter estimates (Nilsson et al., 2017; Dyrby et al., 2013). This type of evaluations establish an upper bound for parameter accuracy in different protocols.

#### 5.6.2 Monte Carlo

In the numerical simulations, the synthetic data is generated using the same model that is used for the fitting. These types of simulations are useful to investigate how diffusion parameters respond to different scenarios such as crossing fibers, partial volume effect (Szczepankiewicz et al., 2015; Alexander et al., 2001; Vos et al., 2011), or degeneracy in parameter estimation (Jelescu et al., 2016; Lampinen et al., 2017, 2018, 2020). There is another type of simulation, Monte Carlo (MC), where the idea is to investigate the model parameters under departure from the model assumptions. In this case, the synthetic data is generated with a procedure that is more complicated than the model that is used for the fitting. MC simulations are especially useful to study complex microstructure (Nilsson et al., 2010; Hall and Alexander, 2009; Balls and Frank, 2009; Ford and Hackney, 1997). In MC simulations, a microstructure substrate is defined numerically and the random walkers move in this environment. For each walker, the signal is predicted using a phase accrued by an ensemble of spins in a simulated gradient waveform. The microstructure substrate can be simulated based on the model assumptions such as parallel cylinders (Nilsson et al., 2010; Hall and Alexander, 2009; Balls and Frank, 2009; Ford and Hackney, 1997). Alternatively, the substrate can be more complicated with more detailed microstructural features such as fiber shape, permeability, undulation, and dispersion (Ong et al., 2008; Nilsson et al., 2010, 2012; Ianuş et al., 2017; Hall and Alexander, 2009). Segmented histology slides yield a complicated substrate in both intra-axonal and extracellular spaces (Xu et al., 2014; Panagiotaki et al., 2010).

#### 5.6.3 Physical phantoms

Physical phantoms represent a simplified version of the tissue and are useful in testing the model with measured data from ideal samples (Fieremans and Lee, 2018). In the process of phantom construction, the ground-truth values of model parameters (ground-truth means one exactly knows what the answer is.) can be controlled and can be measured by microscopy. Phantoms are made of different materials. To have a long life, high reproducibility, and good control over microstructural parameters, some inert materials such as glass or plastic are used in phantom construction. Alternatively, biological phantoms, such as vegetables and cell cultures have a short shelf life but are cheap and easy to prepare but the microstructural features are harder to measure and control. Hollow glass capillaries are utilized to make axon-like phantoms and are useful in verifying diffusion models (Avram et al., 2008; Shemesh et al., 2009), validating the relation between pore size and diffraction pattern from DDE (Shemesh, Özarslan et al., 2010b), testing size estimation with ODE (Li et al., 2014), investigating microscopic anisotropy (Komlosh et al., 2007) and testing dMRI with free gradient waveforms (Siow et al., 2012). Liquid crystals (Topgaard, 2016) as well as phantoms constructed with co-electrospinning (Hubbard et al., 2015; Greiner and Wendorff, 2007), are other examples of axon-like phantoms. Capillaries in asparagus stems and the vascular tissue in celery stalk can model large axons (Lätt et al., 2007; Panagiotaki et al., 2010; Özarslan et al., 2011a; Boujraf et al., 2001) whereas microscopic anisotropy can be studied by asparagus puree (Lasič et al., 2014). Oil-water emulsions can be used to test round cells (Packer and Rees, 1972) as well as compartment models (Topgaard et al., 2002; Håkansson et al., 1998) and multimodal microstructure estimation (Proverbio et al., 2014). Yeast cells can also be used for investigating isotropic intra- and extracellular compartments (Lasič et al., 2011; Silva et al., 2002; Suh et al., 2003; Malmborg et al., 2006). Membrane permeability of the yeast cells change with temperature which can be detected by DDE (Åslund et al., 2009) and the yeast cell sizes range between 4 to 8 micron which makes it ideal for evaluating the cell size estimation methods (Shemesh et al., 2012, 2015). Another manipulatable physical phantom involves human erythrocyte ghosts (Benga et al., 1987), which have been employed in studies on assessing membrane permeability (Benga et al., 1990; Waldeck et al., 1995), origins of non-monoexponential signal decay (Thelwall et al., 2002) and time-dependent diffusion (Özarslan et al., 2006).

#### 5.6.4 Fixed tissue

Measurements on fixed tissue are very close to the *in vivo* and also has many advantages of the phantoms. The disadvantage of this compared to phantoms is that the ground-truth microstructure in the fixed tissue is not as controlled and well-characterized as in phantoms. Obtaining high-quality data from fixed tissue is usually challenging (Dyrby et al., 2011). The time between the death and fixation should be very short (D’Arceuil et al., 2007) but after fixation, the microstructure and diffusion parameters stay stable for several years (Dyrby et al., 2011). However, the fixation process may change the microstructure (Richardson et al., 2014). Another option is using the viable tissues where the bias due to fixation can be avoided (Richardson et al., 2013, 2014; Shepherd et al., 2006). Comparing parameters obtained from dMRI with those from microscopy (Alimi et al., 2020) of the fixed tissue shows that the time-dependent diffusion is in agreement with the restricted diffusion inside axonal compartments (Assaf et al., 2008, Assaf et al., 2000) and also there is a good congruency between the myelinated neurite fractions from dMRI and histology (Jespersen et al., 2010). OGSE-frequency dependence in the intra-axonal and extracellular spaces was observed by Xu et al. (2014). The level of axonal orientation dispersion can be quantified by analysis of the fixed tissue images (Leergaard et al., 2010; Choe et al., 2012).

#### 5.6.5 In vivo + ex vivo

If the same tissue is used for both dMRI and histology, the model parameters, such as axon diameters can be directly validated and interpreted (Barazany et al., 2009). This validation method is impossible in healthy human tissue therefore, comparing with the values reported in the literature can be used as an indirect validation (Alexander et al., 2010). Resection of the tumour can also provide tissue for direct validation (Zetterling et al., 2016; Szczepankiewicz et al., 2016).

Ex vivo optical imaging or staining could provide useful insights for validation of non-invasive techniques like dMRI. Using ex vivo measurements in combination with in vivo dMRI can help in the validation of structural connectivity in the brain. Light microscopy (Howard et al., 2019; Mollink et al., 2017) provides higher spatial resolution while in vivo measurements provide larger 3D fields of view, therefore, the combination of these two is ideal for the mapping of mesoscale connectivity such as subcortical projection systems and layered intracortical projections. With the introduction of complicated q-space sampling schemes, advanced tractography, and fiber orientation distribution reconstruction techniques, the need for validation of these methods is increasing. The overlap of ex vivo dMRI with light microscopy in terms of special resolution in the mesoscopic scale and performing the ex-vivo and in vivo measurement of the same tissue provides the ability to evaluate different microstructural models with a ground-truth measurement. However, ex vivo dMRI data is not usually in high-quality which is sometimes because of the changes that happen in the tissue in the fixation process.

## 6 Signal sensitivity to experimental factors

In this section, we consider various experimental factors typically observed during the acquisition that affect the diffusion signal and subsequent quantitative studies. Among them, the most significant ones are thermal noise, deviations from the prescribed *b*-value, a variable number of diffusion-sensitizing gradients and acquisition shells In terms of signal recovery from a sparse set of gradient directions, there is the SPARC-dMRI challenge (Ning et al., 2015a) as well as relevant methods for sparse recovery (Rathi et al., 2014; Ning et al., 2016). We conclude the section by reviewing alternatives to previously mentioned approaches to quantitatively evaluate the diffusion, which, by definition, should be independent of one or multiple experimental factors. Finally, we give a brief insight into data harmonization techniques and quality assurance protocols that allow comparing and pooling the data from multi-center acquisitions.

### 6.1 Noise in diffusion MRI

Noise in diffusion MRI studies has many faces as it appears under the form of acoustic (Tan et al., 2018), physiological (Walker et al., 2011), and thermal effects (Henkelman, 1985). Acoustic noise is related to inherent sounds generated by the device and received by the patient during the scan. Physiological noise consists of any form of physiological signs such as cardiac or respiratory cycles that induce changes in the brain during scan time. For instance, the cardiac cycle activates changes in cerebral blood flow and cerebral blood volume and induces brain pulsation that affects diffusivity and anisotropy parameters (Nakamura et al., 2009; Brooks et al., 2013). Thermal noise, also called the Johnson–Nyquist noise, originates from the random motion of free electrons in a radio-frequency coil and eddy current losses in the scanned subject or object (Miller and Joseph, 1993; Macovski, 1996; Aja-Fernández and Vegas-Sánchez-Ferrero, 2016). In this paper, however, we mostly focus on thermal noise as it is one of the critical experimental factors that negatively affect diffusion MRI signals. From now on, for simplicity, we will refer to thermal noise, translating to signal fluctuations in the acquired data, exclusively as *noise*.

**Fig. 6:**
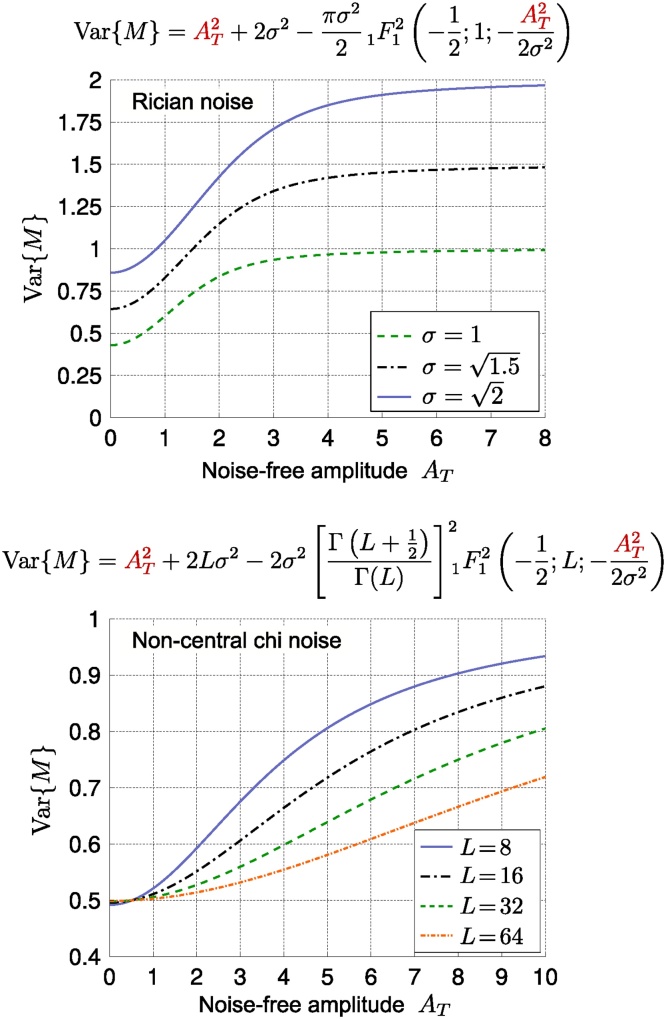
The functional dependence of the variance of Rician and non-central chi (nc-*χ*) distributed random variables *M* in terms of noise-free amplitude signal *A*_*T*_. Variance of nc-*χ* random variable additionally depends on the number of receiver coils *L* and underlying noise standard deviation which is fixed to *σ* = 1 in all cases. The symbol _1_*F*_1_ indicates the confluent hypergeometric function of the first kind while Γ is the gamma function.

In the k-space domain (i.e., acquisition domain) noise is typically assumed to be complex Gaussian distributed with a constant variance of $\sigma_{K}^{2}$ over the acquired data (Henkelman, 1985). As the inverse Fourier transform is a linear and orthogonal operator, the noise propagates to the x-space domain (i.e., spatial domain) preserving its additive Gaussian character. The variance of noise in x-space domain is given then by $\sigma^{2}=|\mathrm{FOV}{|}^{-1}\sigma_{K}^{2}$ with |FOV| being the number of points in the field-of-view (FOV) (Den Dekker and Sijbers, 2014; Aja-Fernández and Vegas-Sánchez-Ferrero, 2016). Once the magnitude data is reconstructed from a (complex) x-space signal representation using a non-linear operator (e.g., the absolute value), the noise is no longer additive, but it follows a signal-dependent nature. In other words, the variance of the noise is in a functional dependence of the hypothetical noise-free amplitude signal. For single-coil acquisitions, the noise is modeled using the well-known Rician distribution as the modulus of the complex Gaussian signal (Rice, 1948, Gudbjartsson and Patz, 1995). In Fig. 6 (top) we show signal-dependency of the variance of Rician distributed signal in terms of the noise-free amplitude signal *A*_*T*_ and fixed noise standard deviation *σ* (i.e., a standard deviation of Gaussian noise in the x-space domain).

**Fig. 7:**
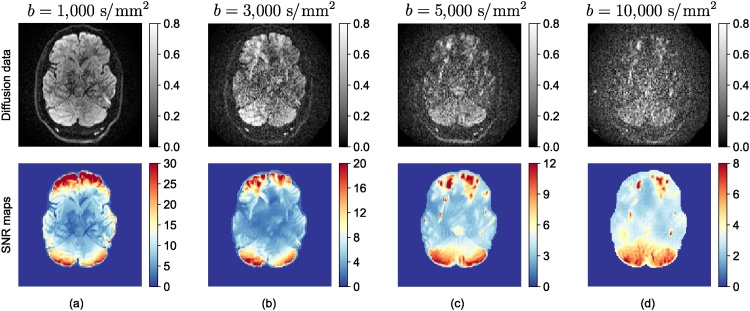
HCP diffusion-weighted brain data for (a) *b* = 1000 s/mm^2^, (b) *b* = 3000 s/mm^2^, (c) *b* = 5000 s/mm^2^ and (d) *b* = 10, 000 s/mm^2^ (top row), and corresponding local signal-to-noise ratio (SNR) obtained with the non-stationary unbiased non-local means filter (Pieciak et al., 2018) and the variance-stabilizing approach (Pieciak et al., 2017). For the sake of visualization, the diffusion-weighted signal has been normalized with baseline signal *S*(0) and the colorbars rescaled to [0–0.8] for all *b*-values.

In parallel acquisitions typically used in diffusion MRI such as the SENSitivity Encoding (SENSE) (Pruessmann et al., 1999; Sotiropoulos et al., 2013; Zhang et al., 2015) and GeneRalized Autocalibrating Partially Parallel Acquisition (GRAPPA) protocols (Griswold et al., 2002; Heidemann et al., 2012; Setsompop et al., 2012), the noise in magnitude data additionally exhibits a non-stationary behavior (Aja-Fernández et al., 2011; Aja-Fernández et al., 2014; Pieciak et al., 2017), i.e., the level of noise changes with the position in the final image *σ*(**x**). This is in contrast to single-coil acquisitions where the level of noise in the magnitude signal is roughly assumed to be constant across the image, i.e., *σ*(**x**) = *σ* (Aja-Fernández et al., 2009). Notice here that a direct consequence of spatially-variant noise in the data is the SNR also changes with position as presented in Fig. 7.

The spatially-variant nature of noise in magnitude data retrieved from parallel accelerated acquisitions depends on various factors such as subsampling ratio in the k-space domain, the number of receiver coils used to acquire the data, correlations between receiver coils and reconstruction procedures used to obtain x-space representation and final magnitude signal (Dietrich et al., 2008; Aja-Fernández et al., 2011; Aja-Fernández et al., 2014). As an illustration, in parallel accelerated SENSE imaging the signal at reach receiver coil in x-space domain is reconstructed using the inverse Fourier transform. Noise in the x-space domain increases then proportionally with the subsampling ratio *r* as given by Aja-Fernández et al. (2014)

(12) $$\sigma_{l}^{2}=\frac{r}{\left|\mathrm{FOV}\right|}\sigma_{K_{l}}^{2},\;\;\;l=1,\ldots ,L,$$

where $\sigma_{K_{l}}^{2}$ and $\sigma_{l}^{2}$ are underlying noise variances at *l*th receiver coil in k-space and x-space domains, respectively, and *L* is the number of receiver coils. Once the magnitude signal is reconstructed using the (weighted) least-squares procedure following by an absolute operator it presents a non-stationary signal-dependent Rician noise (Aja-Fernández et al., 2014; Pieciak et al., 2017). In other words, the noise besides of being a signal-dependent one, as in single-coil acquisitions, changes also its variance with the position.

Another statistic frequently used in diffusion MRI is the non-stationary non-central chi (nc-*χ*) model. The nc-*χ* statistic originates from squared sum-of-squares (SoS) of Gaussian random variables, all assumed to have the same distributional parameters. Therefore, the nc-*χ* model is suited to present the noise in Cartesian GRAPPA+SoS acquisitions as the final magnitude data is retrieved using the SoS procedure from *L* complex samples once no correlations are assumed between them (i.e., coils are assumed to be non-correlated) (Constantinides et al., 1997; Aja-Fernández et al., 2011). Over here, the noise in the magnitude data also presents a signal-dependent nature (Fig. 6; bottom), and its properties change with spatial position. However, to properly address noise characteristics in the magnitude GRAPPA + SoS signal retrieved from correlated data, as typically observed in real scenarios, it is necessary to employ a parametrized nc-*χ* model with *effective* parameters namely the increased variance of noise $\sigma_{\mathrm{eff}}^{2}(\text{x})$ and decreased number of receiver coils *L*_eff_(**x**) (Aja-Fernández et al., 2011; Aja-Fernández et al., 2013). Secondary to SoS reconstruction in GRAPPA is that magnitude data can also be obtained with a spatially matched filter (Walsh et al., 2000). In this scenario, the final magnitude signal follows a signal-dependent non-stationary Rician noise.

To conclude, the noise in magnitude diffusion MRI data obtained from parallel accelerated techniques is signal-dependent and follows a non-stationary behavior depending on the acquisition set-up and data reconstruction algorithm. In SENSE imaging the noise characteristics are fully represented with spatially-variant noise level map *σ*(**x**). In GRAPPA+SoS the noise must be specified by two effective spatially-variant parameters that is to say the variance $\sigma_{\mathrm{eff}}^{2}(\text{x})$ and number of coils *L*_eff_(**x**).

### 6.2 Consequences of noise and ways of dealing with it

Noise in medical imaging is typically interpreted as an observable quality deterioration of the data. In diffusion MRI, however, the noise has far-reaching consequences as it affects the fitting procedures and therefore translates to quantitative measures such as the ones derived from DT-MRI, DKI, or MAP-MRI.

In recent years, several methods have been proposed to mitigate the unfavorable effects of the non-stationary Rician and nc-*χ* noise. These include well-celebrated the non-local means framework that evaluates the similarity in terms of non-local patches (Manjón et al., 2010, Manjón et al., 2013, Manjón et al., 2015, Bouhrara et al., 2016, Sudeep et al., 2018, Pieciak et al., 2018), the random matrix theory approach which exploits the Marchenko–Pastur law of the eigenvalues of noise (Veraart et al., 2016c) and a group of algorithms that uses joint information from spatial and *q*-space domains to significantly improve previous results (St-Jean et al., 2016, Chen et al., 2019b, Chen et al., 2019a). Notice here that any aggregation-based algorithm introduces a systematic bias to aggregated signal that should be corrected prior to a quantitative interpretation. Such methodologies have been proposed for averaged (stationary) Rician/nc-*χ* signals including a correction in squared magnitude domain (McGibney and Smith, 1993, Miller and Joseph, 1993, Aja-Fernández and Krissian, 2008, Wiest-Daesslé et al., 2008) or the so-called *analytically exact correction scheme* using a fixed point formula of SNR (Koay and Basser, 2006). Recently, Pieciak et al. (2018) derived new closed-form formulas for aggregation of squared and non-squared non-stationary Rician and nc-*χ* signals. These formulas can be used along with any averaging-based filters such as the aforementioned non-local means or bilateral filters to correct noise-induced bias in non-stationary data. Contrary to the magnitude space noise removal algorithms, some authors have proposed removing noise in complex x-space domain (Wirestam et al., 2006, Baselice et al., 2017, Cordero-Grande et al., 2019). Such approaches provide unbiased results since they operate directly on a Gaussian distributed signal before the magnitude reconstruction is performed.

As the noise in magnitude signal retrieved from accelerated diffusion MRI acquisitions follows a non-stationary form, a proper spatially-variant noise estimation technique must be arranged to feed the adaptive denoising algorithm. Such techniques have also been recently introduced both for non-stationary Rician (Maximov et al., 2012, Veraart et al., 2013a, Aja-Fernández et al., 2015, Pieciak et al., 2017) and non-stationary nc-*χ* noise (Tabelow et al., 2015, Veraart et al., 2016b, Pieciak et al., 2016). Unlike the variance-stabilizing approach (VST; Pieciak et al., 2016, Pieciak et al., 2019) that firstly transforms the signal-dependent Rician/nc-*χ* noise to a signal-independent variate and then estimates the noise pattern, most approaches estimate noise maps assuming Gaussian distribution and then apply a *post-hoc* correction by Koay and Basser (2006). As a side note, most of the methods intended for diffusion MRI estimate the spatially-variant noise map given all gradient directions at a certain *b*-value. In other words, a single noise map is produced independently from the number of gradients from the same acquisition shell (see Veraart et al., 2016b). On the other hand, a group of methods designed to work on a single-slice can estimate noise patterns separately for each diffusion-sensitizing gradient direction. This is done without any additional information needed, such as the repeated acquisitions, sensitivity maps in the case of SENSE or reconstruction coefficients for GRAPPA (see Aja-Fernández et al., 2015, Tabelow et al., 2015, Pieciak et al., 2016, Pieciak et al., 2017). All in all, both approaches to noise estimation provide excellent results and can be successfully used in diffusion MRI studies.

### 6.3 Sensitivity to changes in the number of gradients

Proper sampling of the q-space domain in diffusion MRI plays a crucial role in the optimization process of data acquisition procedures. In DT-MRI, various acquisition schemes and side effects due to protocol changes have been exhaustively studied in the literature so far (Papadakis et al., 1999, Skare et al., 2000, Jones, 2004, Landman et al., 2007, Barrio-Arranz et al., 2015). Early efforts by Papadakis et al. (1999) and Skare et al. (2000) investigated possible advantages in retrieving anisotropy measures once going beyond the standard six measurements protocol. A more comprehensive study on the effects of gradient sampling by Jones (2004) has shown that at least 20 non-collinear and non-coplanar diffusion-sensitizing gradient directions are required for robust estimation of fractional anisotropy. In contrast to anisotropy measure, at least 30 gradient directions are needed to obtain the mean diffusivity and tensor orientation.

Veraart et al. (2013b) showed that increasing the number of gradient directions translates differently to quantitative descriptors depending on the method used to estimate the tensor. In general, the performance of the WLS (Weighted Least Squares) estimator depends on the selection of the weighting mechanism. For instance, the multi-step approaches (see Veraart et al., 2013b for details) appear to be more robust than the original proposal by Basser et al. (1994a). Precisely, increasing the number of gradient directions improves the accuracy of retrieved quantitative maps obtained via the multi-step WLS approach with the least-squares method used as a pre-estimation step. However, once applied the squared noisy signals as a weighting mechanism, the results are biased, i.e., a negative bias is observed with FA and MD, whereas a positive one with the MK. Contrary to linear estimators, the non-linear least-squares estimator (Koay et al., 2006) introduces a constant bias for the FA and an increasing bias for the MD and MK parameters as the number of gradients increases (Veraart et al., 2013b).

The evaluation of the optimal number of gradients in higher-order models is not as straightforward as in DT-MRI or DKI. Here, not only the reconstruction technique might affect the analysis, but many other factors, such as the order of harmonics used to represent the data *l* (Tournier et al., 2013, Schilling et al., 2017), regularization constants (Fick et al., 2016) and other tunable method-dependent control parameters (Hutchinson et al., 2017). Moreover, the algorithms might provide either continuous or discrete information (Tournier et al., 2013). All these factors make it challenging to identify a single number of gradient directions that meet the criteria of all High Angular Resolution Diffusion Imaging (HARDI) methods. However, in Tournier et al. (2013), the authors tried to find a consensus and determined the optimal number of different orientations for robust Q-ball imaging and spherical harmonic deconvolution. This methodology uses the angular properties of the signal itself analogous to the well-known Nyquist–Shannon theorem (Shannon, 1949). Once fixing the order of harmonic degree *l* in the Spherical Harmonics (SHs) decomposition, it sets an upper limit on the angular frequencies that can be resolved. In clinical conditions, harmonic degree equals *l* = 8 has been found to enable characterizing angular properties of the signal. Consequently, at least 45 gradient directions (no. of gradients = (*l* + 1)(*l* + 2)/2) are necessary to resolve the angular features of the signal. Further substantial increase in the number of measurements does not involve notable improvements in the angular properties of the signal. However, due to imperfections in sampling over the sphere, a slightly higher number of gradients is typically favored (Tournier et al., 2013).

The optimal number of gradient directions may also depend on other factors, such as spatial resolution or the *b*-value. In Li et al. (2018), the authors propose a linear formula in terms of the *b*-value to determine the minimal number of diffusion-sensitizing gradients that enabled robust measurement of spherical mean signal. Here, for a common SNR ∼ 20 measured at the baseline signal, the number of gradients can be calculated using the formula no . of gradients = 10 × *b*/*b*_1_ (*b*_1_ = 1000 s/mm^2^). In the signal representations category, the reduction of the number of encoding directions in the MAP-MRI seems not to affect the EAP measures significantly at *b*_max_ = 6000 s/mm^2^ (Avram et al., 2016). This study was not confirmed by Fick et al. (2016), where significant deteriorations have been observed with the RTOP and RTAP. Further, Pieciak et al. (2019) has shown that the relative error of the RTOP measure increases approximately linearly with decreasing the number of gradients once fixing the maximal *b*-value parameter.

### 6.4 Sensitivity to changes in the number of shells and *b*-values

The number of acquisition shells (i.e., distinct *b*-values) is yet another factor that seems to be one of the most critical ones in diffusion imaging. The optimal *b*-value(s) and/or the number of shells depend on the modality to be used by the operator and is a topic of vigorous debate in the community (Kingsley and Monahan, 2004, Jones, 2007, Tournier et al., 2013, Chuhutin et al., 2017, Hutchinson et al., 2017, Peña-Nogales et al., 2020). In general, higher *b*-value (i.e., setting larger *q*-value leaving the diffusion time constant) causes the acquisition process more vulnerable to catch smaller particle motions. On the other hand, the SNR of collected data drops down as the *b*-value increases (see Fig. 7). Therefore, more total number of measurements are typically favored at high *b*-value acquisitions.

The selection of the optimal number of shells and *b*-values depends on the acquisition goal and planned diffusion MRI techniques to be used. In the DT-MRI adult brain studies a typical set-up is a single-shell acquisition with *b*-value near 1000 s/mm^2^ while in neonate and infant brains the *b*-value at 700 s/mm^2^ is recommended due to higher water content (O’Donnell and Westin, 2011, Pannek et al., 2012). In DKI, the situation is more complex as the optimal maximal *b*-value seems to depend on a tissue type to be quantified. For example, the *b*-value of about 2500 s/mm^2^ has been found to be optimal in WM areas while *b*-values less than 1000 s/mm^2^ achieves the minimum error in GM regions (Chuhutin et al., 2017). Higher *b*-values in the range of 2000–4000 s/mm^2^ are satisfactory for spherical deconvolution (Tournier et al., 2008, Tournier et al., 2013, Schilling et al., 2017), mixture models such as the multi-tensor (Tuch et al., 2002), Q-Ball imaging (Kuo et al., 2008) or NODDI (Parvathaneni et al., 2018).

Even higher maximal *b*-values about 6000 s/mm^2^ are beneficial for clinical applications using the MAP-MRI framework (Avram et al., 2016). Just as importantly, most of the studies on optimal *b*-value(s) are purely empirical as they evaluate a set of configurations and select the optimal set-up in terms of precision and accuracy of derived quantitative parameters. Recently, Peña-Nogales et al. (2020) proposed a new framework to obtain a set of optimal *b*-values for the Apparent Diffusion Coefficient (ADC) imaging in the liver using a Cramér–Rao lower bound based analysis under Rician noise distribution.

Altering the *b*-value typically influences parametric signal representation and diffusion MRI studies. For example, Hutchinson et al. (2017) observed significant changes in the mean kurtosis (MK) and fractional anisotropy (KFA) under a varying number of shells and maximal *b*-value. In particular, the KFA seems to be highly positively biased when the maximal *b*-value decreases leading to a lack of distinction between WM and GM areas. Tournier et al. (2013) has shown a functional dependence of the spherical harmonics (SHs) coefficients on the *b*-value parameter in q-ball imaging and spherical deconvolution, i.e., the reconstructed signal profile becomes sharper as the *b*-value increases. In practice, the acquisition at *b* = 3000 s/mm^2^ with harmonic degree set to *l* = 8 is enough to capture the angular properties of the diffusion signal with both techniques. Notice here that the dependence of signal profile on the *b*-value had been revealed earlier for two diffusion-tensor compartments by Alexander et al. (2001). Concerning the Q-ball imaging, Schilling et al. (2017) observed that increasing the *b*-value parameter results in an enlarged estimate of crossing fiber fraction in the WM voxels independently of the SHs order used to reconstruct the orientation distribution functions (ODFs).

**Fig. 8:**
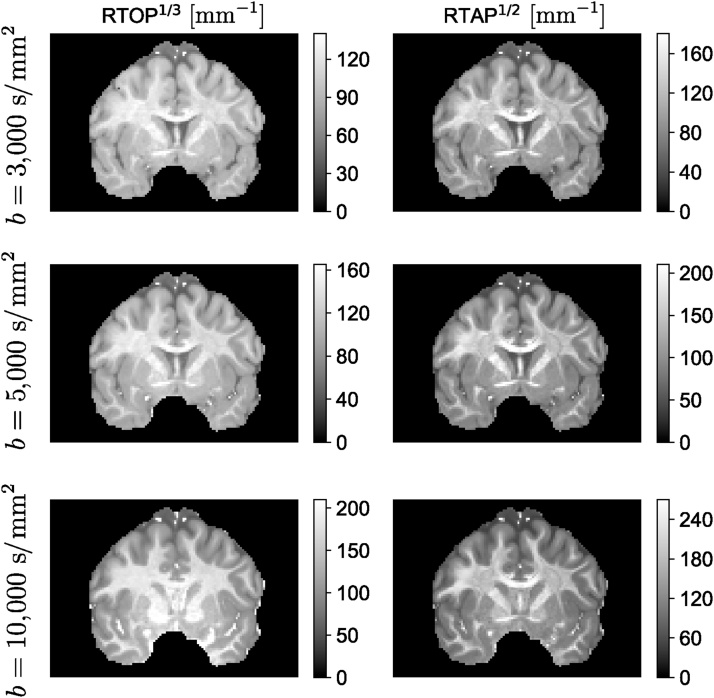
Comparison of RTOP and RTAP measures derived from the MAPL framework (Fick et al., 2016) under different maximal *b*-value parameter. The RTOP measures have been scaled to the power of 1/3 while the RTAP to the power of 1/2.

In multi-shell acquisitions, various studies have also examined how the changes in maximal *b*-value and number of shells affect the precision and reproducibility of quantitative metrics. For instance, in the Laplacian MAP-MRI (MAPL) technique, Fick et al. (2016) observed increased estimates of apparent axon diameter and decreased mean value of non-Gaussianity parameter over the corpus callosum when reducing the maximal *b*-value. When compared to histological studies, the apparent axon diameter seems to be overestimated even at the order of magnitude. This is likely due to the relatively moderate value of the peak gradient strength used to acquire the HCP MGH data, i.e., 100 mT/m (Van Essen et al., 2012). Microstructural-related measures obtained from the MAP-MRI framework, such as the RTOP, RTAP, or RTPP, were observed to be rather stable across a different number of shells and maximal *b*-value (Hutchinson et al., 2017). However, these results are in contradiction to a recent paper by Aja-Fernández et al. (2020), where a systematic functional dependence of all three parameters with an increasing number of shells and maximal *b*-value has been observed for the HCP MGH data. Most notably, significant overestimations in the EAP-derived microstructural indices have been recognized when including the fourth shell at *b* = 10, 000 s/mm^2^. This unstable behavior of the quantities across the maximal *b*-value has been observed despite the methodology used to calculate the EAP function, as verified with the MAP-MRI (Özarslan et al., 2013), MAPL (Fick et al., 2016), and Radial Basis Functions (Ning et al., 2015b). In Fig. 8 we show the RTOP and RTAP measures obtained from the MAPL framework on an axial slice of HCP MGH data. The quantities have been calculated using three different maximal *b*-values particularly *b* = 3000 s/mm^2^, *b* = 5000 s/mm^2^ and *b* = 10, 000 s/mm^2^ corresponding to two-, three- and four-shells acquisition, respectively.

In Table 4 we summarize some recent studies on experimental factors and their possible consequences in non-Gaussian diffusion MRI.

**Table 4:** The summary of recent studies on experimental factors and possible alterations in non-Gaussian diffusion MRI across different modalities.

Author	Modality	Factors	Relevant reported effects
(Tournier et al., 2013)	QBI, SD	Gradients, *b*-value	The acquisition with the *b*-value approximately at 3000 s/mm^2^ and SHs degree *l* = 8 maximizes the achievable angular resolution, and at least 45 diffusion-sensitizing gradient directions are required to capture the angular properties of the signal. Further substantial increase in the number of gradient directions does not improve the angular properties of the signal. The angular information for *l* > 8 at *b* = 3000 s/mm^2^ is negligible
(Veraart et al., 2013b)	DKI	Gradients, SNR	The accuracy of the MK depends on the method used to estimate the kurtosis tensor and the choice of the weights in the case of the WLS estimation. Increasing the number of gradients (1) reduces the bias of the MK measure in the WLS multi-step approach, and (2) enlarges the bias of the MK measure when using standard WLS (Basser et al., 1994a) and NLS procedures (Koay et al., 2006))
(Hosseinbor et al., 2013)	BFOR, SPFI, mq-DPI	SNR	Decreasing the SNR of the signal leads to mixed effects in changes of RTOP and GFA indices (i.e., overestimation and underestimation) depending on the properties of the measured component (e.g., fast/slow isotropic, fast/slow anisotropic)
(Ning et al., 2015b)	RBFs, 3D-SHORE	Gradients	Decreasing the number of gradient directions increases the percentage of false peaks in two fiber crossing regions
(Fick et al., 2016)	MAPL	Shells, gradients, *b*-value	Decreasing the *b*-value parameter (the number of shells and diffusion-sensitizing gradients as well) leads to an augmentation of the apparent axon diameter and reduction of the mean value of the non-Gaussianity NG parameter both in the Corpus Callosum region
(Avram et al., 2016)	MAP-MRI	Noise	Decreased the SNR (i.e., increased noise level) of the signal translates to (1) overestimations in the RTOP, RTAP, RTPP, NG (including NG_∣∣_, and NG_⊥_) and (2) underestimations in the PA, all in WM regions. The measures derived from projecting the propagator on axes (i.e., RTAP, NG_∣∣_) and planes (RTPP, NG_⊥_) of the anatomical coordinate system are more prone to noise than overall EAP-derived quantities (i.e., RTOP, NG)
(Schilling et al., 2017)	QBI	Gradients, *b*-value	Decreasing the number of diffusion-sensitizing gradients translates to (1) a positive bias in the GFA (up to about 30% deviation for reduced acquisition scheme from 90 to 48 gradients and the SH order 8 fit), (2) the overestimation of the number of fibers (peaks) inside a single voxel independently of the SHs order and (3) a positive (negative) angular bias in the ODF up to +15^∘^ (−10^∘^) at angles less than 60^∘^ (between 80^∘^ and 90^∘^) for reduced acquisition from 90 to 48 gradients and the SH order 8 fit. Increasing the *b*-value parameter (from *b* = 1000 s/mm^2^ to *b* = 3000 s/mm^2^) results in increased estimate of crossing fiber fraction in the WM voxels independently of the used SHs order
(Hutchinson et al., 2017)	DKI, MAP-MRI, NODDI	Shells, gradients, *b*-values, noise	Changes in maximal *b*-value (i.e., number of shells and gradients) lead to significant alterations in the MK, KFA, NG, and PA quantities (e.g., the KFA is highly positively biased). The noise causes (1) a considerable amplification of NG, intracellular volume fraction *V*_IC_ and PA, and (2) mixed changes in histogram and mode of the MK and isotropic volume fraction *V*_ISO_
(Chuhutin et al., 2017)	DKI	*b*-value	Decreasing the maximal *b*-value leads to the increased value of the MK parameter
(Parvathaneni et al., 2018)	NODDI + AMICO	Gradients, *b*-value	The recommended acquisition set-up for NODDI derived parameters is the *b*-value at 2500 s/mm^2^ with at least 128 total gradient directions.
(Li et al., 2018)	SMT	gradients	The recommended number of uniformly distributed diffusion-sensitizing gradients defined in terms of CV < 0.05 to a reference is given by 10 × *b*/*b*_1_ (*b*_1_ = 1000 s/mm^2^) for a typical SNR ∼ 20 measured at the baseline. Decreasing the number of gradients increases at an exponential rate the CV of the signal
(Pieciak et al., 2019)	MAP-MRI, MAPL	Gradients	Decreasing the number of diffusion-sensitizing gradients increases the mean relative error (up to 20% for 10% measurements out of 270) and the standard deviation of the RTOP both for the MAP-MRI and MAPL. The mean relative error of the RTOP increases approximately linearly in the function of the subsampled gradients
(Aja-Fernández et al., 2020)	MAP-MRI, MAPL, RBFs	Shells, gradients, *b*-value	Increasing the maximal *b*-value parameter (i.e., number of shells and gradients) leads to the amplification of microstructural-related measures (RTOP, RTAP, RTPP) independently of the technique used to estimate the EAP function (MAP-MRI, MAPL, RBFs)

**Modalities:** QBI – Q-ball imaging, SD – spherical deconvolution, NODDI – neurite orientation dispersion and density imaging, DKI – diffusion kurtosis imaging, 3CM – three-compartment model, SMT – spherical mean technique, MAP-MRI – mean apparent propagator MRI, MAPL – Laplacian MAP, RBFs – radial basis functions, 3D-SHORE – three-dimensional simple harmonic oscillator based reconstruction and estimation, AMICO – accelerated microstructure imaging via convex optimization.

**Measures or properties:** MK – mean kurtosis, GFA – generalized fractional anisotropy, ODF – orientation distribution function, MK – mean kurtosis, KFA – kurtosis fractional anisotropy, NG – non-Gaussianity, PA – propagator anisotropy, RTOP – return-to-the-origin probability, RTAP – return-to-the-axis probability, RTPP – return-to-the-plane probability, EAP – ensemble average propagator.

**Others:** SH – spherical harmonics, WLS – weighted least squares, NLS – non-linear least squares, SNR – signal-to-noise ratio, WM – white matter, CV – coefficient of variation.

As unveiled in this section, the optimal *b*-value(s) set-up depends on many factors, including the modality to be used, fitting procedure, tissue, and the study group to be imaged (i.e., neonates, infants or adults). Thus, any diffusion MRI data collection should be preceded by a systematic literature review in order to choose the optimal acquisition protocol.

### 6.5 Managing experimental factors

As highlighted in earlier sections, numerous experimental factors interfere with diffusion MRI studies to a varying extent. Therefore, considerable care must be taken when analyzing the data collected under various acquisition conditions. The solutions to address the unwanted experimental effects fall into two categories, particularly the techniques that calculate unbiased measures and data harmonization protocols.

The former group includes alternative measures that can be obtained for example from the q-space domain data representation (Wu and Alexander, 2007, Wu et al., 2008, Aja-Fernández et al., 2018, Pieciak et al., 2019, Aja-Fernández et al., 2019, Aja-Fernández et al., 2020). Illustrative examples are the average sample diffusion (ASD) and the diffusion coefficient of variation (CVD). Both measures, by definition, are insensitive to changes in the number of diffusion gradient directions and the *b*-value (Aja-Fernández et al., 2018). These two indices are defined directly in the q-space domain, and for regularly sampled data over the sphere can be expressed as follows:

(13) $$\mathrm{ASD}=-\frac{1}{b}\frac{1}{N_{g}}\sum_{i=1}^{N_{g}}\log E({\text{q}}_{i}),$$

(14) $$\mathrm{CVD}=\sqrt{\frac{V(\log E({\text{q}}_{i}))}{\frac{1}{N_{g}}\sum_{i=1}^{N_{g}}{\left(\log E({\text{q}}_{i})\right)}^{2}}},$$

where *E*(**q**_*i*_) = *S*(*b* ; **x**_*i*_)/*S*(*b* = 0 ; **x**_*i*_) is the normalized diffusion-weighted signal, **x**_*i*_ is the spatial location in *i*-th direction, *N*_*g*_ is the number of diffusion-sensitizing gradients and V(·) is the sample variance.

**Fig. 9:**
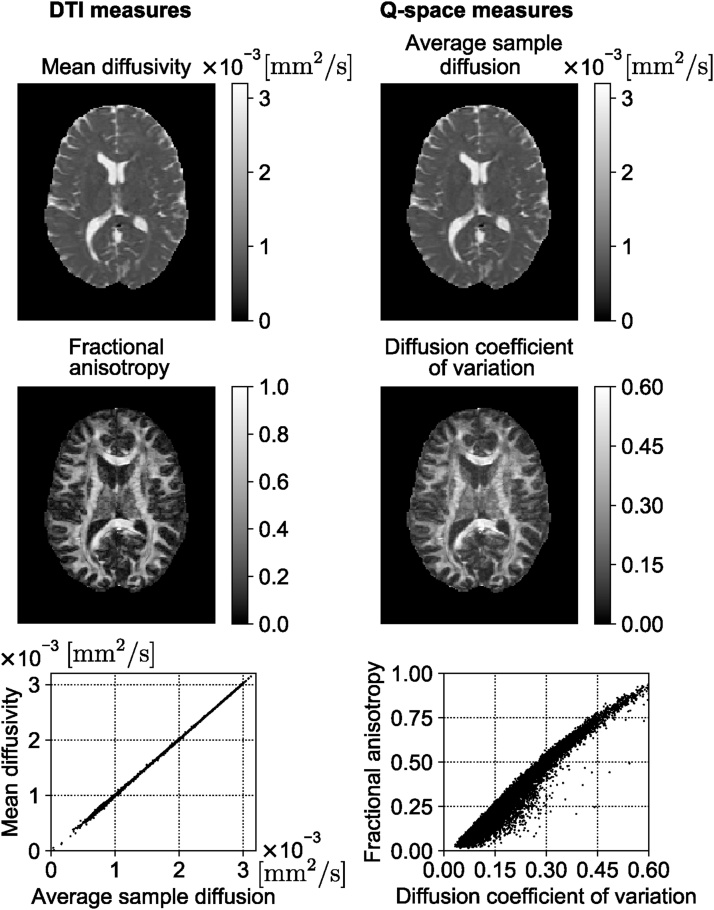
Comparison of DT-MRI-based mean diffusivity to the average sample diffusion measure (ASD) and fractional anisotropy to the diffusion coefficient of variation (CVD) all obtained from 30 diffusion-sensitizing gradient directions and *b* = 1000 s/mm^2^. The ASD and CVD measures were derived directly from the q-space data representation. The Pearson correlation coefficients equal 0.99988 and 0.97501 for diffusivity and anisotropy measures, respectively.

The ASD measure provides a substitute for the mean diffusivity. The CVD tells about the variate of diffusion inside a single voxel, thus can be related to the fractional anisotropy. In Fig. 9, we compare the DT-MRI-related quantities to the above-mentioned unbiased measures derived directly from the q-space representation of the signal at *b* = 1000 s/mm^2^. Both q-space measures exhibit high correlations expressed by the Pearson correlation coefficient, to the DT-MRI based parameters over the foreground area.

The alternative measures should exhibit unbiasedness in terms of one or multiple factors such as the noise (e.g., the expectation of log-Rician random variable is assumed to be unbiased for high SNR; see Aja-Fernández et al., 2018), the number of diffusion-sensitizing gradients or the *b*-value. Besides, they should provide new diagnostic information (see Aja-Fernández et al., 2019) previously hindered by other techniques or exhibit high correlations to community accepted quantities for instance DT-MRI-based parameters (Aja-Fernández et al., 2018) or microstructural quantities derived from the MAP-MRI model like the RTOP, RTAP, and RTPP (Pieciak et al., 2019, Aja-Fernández et al., 2020).

The latter group includes data harmonization methods. Under the term *data harmonization*, we define any mathematical algorithm or statistical tool that transforms the data to suppress the experimental factors that arose from diverse data acquisition protocols or centers (Zhu et al., 2018, Huynh et al., 2019a, Mirzaalian et al., 2016). Data harmonization enables us to compare and pool the data acquired across multiple centers and thus to evaluate rare cases not always geographically available to be scanned in the same place. Moreover, data harmonization protocols allow testing if the found effects are seen in a single cohort, or they are regularly observed in the population (Zhu et al., 2018).

Diffusion MRI data collected from various scanners may vary in many aspects including the vendor used to scan the subject or object, acquisition set-up (e.g., sequence, number, and distribution of diffusion-sensitizing gradients, *b*-value(s), etc.) and computational algorithms used to reconstruct the data (e.g., GRAPPA, SENSE and their modifications). In the intra-scanner session, one also observes inherent scanner instabilities, such as the static magnetic field inhomogeneity and eddy currents distortions (Bruce et al., 2018) or Gibbs ringing artifacts (Perrone et al., 2015, Veraart et al., 2016a). Besides, the data before the analysis might be corrected using different non-standardized algorithms.

In recent years, various quality assurance protocols for multi-center studies have been proposed (Belli et al., 2016, Zhou et al., 2017). These procedures enable to assess the reliability of diffusion MRI metrics across scanners before initiating any multifaceted studies. For instance, Helmer et al. (2016) proposed a histogram distance-based method to explore within- and between site effects. This procedure allows verifying whether the data coming from intra- and inter-scanner trials with different vendors and acquisition parameters stay in acceptable limits.

To harmonize the data, we can recognize parametric (Mirzaalian et al., 2016, Huynh et al., 2019a, Karayumak et al., 2019) and deep learning-based algorithms (Nath et al., 2018, Nath et al., 2019). For instance, Mirzaalian et al. (2016) proposed the multi-site harmonization pipeline that enables to correct differences between different scanners. To this end, they register each diffusion volume to a common template, convert diffusion MRI signal to the SH basis and calculate rotation invariant spherical harmonic features followed by a voxelwise correction. This correction is done by scaling each SH energy band with the known energy band difference between each scanner. Different work by Huynh et al. (2019a) harmonizes diffusion MRI data directly in the space of diffusion-weighted signal via the method of moments estimating a linear mapping function.

All in all, many factors may lead to variabilities in inter- and intra-scanner diffusion MRI scans both at the acquisition set-up and data preprocessing levels. All these factors make multi-center comparison and data pooling difficult, introducing a certain degree of variability to the analysis. As a response to weak statistical power and reproducibility of neuroimaging studies, the community has started developing diffusion MRI data harmonization protocols.

## 7 Conclusion

Despite the great amount of information that we get from microstructure imaging, the available biophysical models are oversimplified. There are simplifying assumptions such as fixing the diffusivities, fixing the axon diameter distribution, neglecting the effect of exchange, perfectly aligned cylinders, tortuosity constraint, and so on. These simplifications may bias the estimation of the remaining parameters. Adding more parameters to the model makes fitting more complicated and unstable. The recent trend toward computational models may help to capture the effects of undulation, exchange, branching, and tortuosity (Nedjati-Gilani et al., 2017, Silva et al., 2002).

Most published works have collected data using the SDE approach, however advanced acquisition methods may increase the sensitivity to tissue features. There are clear benefits to using special sequences. For example, the OGSE improves the sensitivity to axon diameter in the presence of orientation dispersion (Drobnjak et al., 2016). DDE and QTE can disentangle microscopic anisotropy from distributed microscopic pore size which we cannot distinguish using simple SDE (Mitra, 1995, Özarslan and Basser, 2008, Özarslan, 2009, Jespersen et al., 2013, Lasič et al., 2014, Szczepankiewicz et al., 2016). The DDE improves the sensitivity to exchange (Callaghan and Furó, 2004, Nilsson et al., 2013, Lasič et al., 2011). Using more reliable methods of fitting improves the precision of the parameter maps. The dictionary-based approaches are faster than nonlinear methods and are good for the estimation of parameters if we have a large database of images.

Except for the sequence and representation or model selection to be used of no lesser importance are other factors that can interfere with diffusion signal and quantitative studies. The noise in diffusion signal magnitude is signal-dependent and in parallel acquisitions, it typically follows a non-stationary behaviour (Tabelow et al., 2015, Aja-Fernández and Vegas-Sánchez-Ferrero, 2016, Pieciak et al., 2017). Hence, a proper spatially-variant noise estimation algorithm (see Tabelow et al., 2015, Aja-Fernández et al., 2015, Veraart et al., 2016b, Pieciak et al., 2016, Pieciak et al., 2017) should be used to feed the adaptive filtering algorithm (Veraart et al., 2016c, St-Jean et al., 2016, Pieciak et al., 2018, Chen et al., 2019a). To that end, considerable attention must be paid when choosing a proper denoising algorithm as contrary to structural imaging; any noise-induced bias can alter the quantitative studies (Jones and Basser, 2004, Lauzon et al., 2013, Gahm et al., 2014, Pizzolato et al., 2016, Gilani and Johnson, 2019, Pieciak et al., 2018).

Numerous experimental factors might affect the diffusion signal with particular attention to changes in the number of gradient directions, acquisition shells, *b*-value(s), and the SNR of the signal. Extreme caution must be used when choosing the estimation approach in the DT-MRI as the classical WLS approach, and NLS might introduce a systematic bias to the measures when increasing the number of gradient directions (Veraart et al., 2013b). In HARDI methods, at least 45 gradient directions with the SHs harmonic degree at *l* = 8 are required to retrieve the angular properties of the signal (Tournier et al., 2013). Experimental factors can significantly alter the diffusion signal, e.g., decreasing the number of gradients introduces a positive bias to the GFA (Schilling et al., 2017) and linearly increases the mean relative error to the RTOP (Pieciak et al., 2019), changes in the number of shells (gradients and the *b*-value) is critical to the quantitative measures including the KFA, NG, PA, RTOP, RTAP, RTPP, and apparent axon diameter estimation (Hutchinson et al., 2017, Aja-Fernández et al., 2020, Fick et al., 2016), the low SNR affect the microstructural (i.e., RTOP, RTAP, RTPP) and non-Gaussianity (NG, NG_∣∣_, NG_⊥_) measures (Avram et al., 2016). To deal with experimental factors one can use either the unbiased measures defined directly over the q-space domain (Wu et al., 2008, Aja-Fernández et al., 2018, Pieciak et al., 2019, Aja-Fernández et al., 2020) or employ harmonization protocols to pool the data across multiple sources (Mirzaalian et al., 2016, Huynh et al., 2019a).

## Author contributions

**Maryam Afzali:** Conceptualization, writing – original draft preparation, Investigation. **Tomasz Pieciak:** Conceptualization, writing – original draft preparation, investigation, visualization. **Sharlene Newman:** Writing – reviewing and editing. **Eleftherios Garifallidis:** Writing – reviewing and editing. **Evren Özarslan:** Conceptualization, writing – reviewing and editing, validation, investigation. **Hu Cheng:** Writing – reviewing and editing. **Derek K Jones:** Writing – reviewing and editing, validation, supervision.

## Declarations of interest

None.

## Footnotes

1 The stick model refers to neurites as cylinders of infinitesimal diameter (Behrens et al., 2003). See Section 5.1.
